# Vitality insights of fish escaping from a sorting grid installed on a bottom trawl net

**DOI:** 10.1038/s41598-024-84364-6

**Published:** 2025-01-02

**Authors:** Andrea Petetta, Bent Herrmann, Daniel Li Veli, Giovanni Canduci, Ivan Tatone, Sara Bonanomi, Lorenzo Jacopo De Santis, Giordano Giuliani, Massimo Virgili, Paolo Carpentieri, Alessandro Lucchetti

**Affiliations:** 1 Institute for Marine Biological Resources and Biotechnology (IRBIM), National Research Council (CNR), Largo Fiera della Pesca 2, 60125 Ancona, Italy; 2 The Arctic University of Norway UIT, Hansine Hansens veg 18, 9019 Tromsø, Norway; 3 Fishing Gear Technology, SINTEF Ocean, Trondheim, Norway; 4 National Institute of Aquatic Resources, Technical University of Denmark, Hirtshals, Denmark; 5 CoNISMa, National Inter-University Consortium for Marine Science, Rome, Italy; 6 General Fisheries Commission for the Mediterranean (GFCM), Food and Agriculture Organization of the United Nations (FAO), Rome, Italy

**Keywords:** Discard reduction, Bottom trawl, Juveniles’ sorting grid, Survival estimates, Bycatch reduction device, Mediterranean demersal fisheries, Environmental impact, Marine biology, Conservation biology

## Abstract

Sorting grids to exclude the juveniles of species targeted by bottom trawl fisheries from the catch are among the most promising solutions to reduce discards. We tested a two-sections Juveniles’ Sorting Grid (JSG) in a Mediterranean fisheries restricted area. First, we provided information on the vitality of individuals escaping from the JSG bars during towing, by analysing underwater footage. Then, we evaluated the catch performance of the JSG-equipped trawl compared to a standard trawl by analysing both the full species community in the catches and the main commercial species. The probability for individuals to be alive while escaping from JSG was always higher than 65% (on average), with some species (e.g. red mullet, gurnards, 91–99% on average) showing significantly higher probability than others (e.g. European hake, crustaceans, 65–82% on average). The installation of a JSG in the trawl net did not change the overall catch composition in the codend, although significant differences were observed at the single species level. The JSG was effective at reducing undersized individuals of European hake, although a loss of legal-sized individuals was observed due to escapement. A significantly lower retention of the JSG-equipped trawl was also observed for other commercial species, such as deep-water rose shrimp and broadtail shortfin squid.

**Supplementary Information:**

The online version contains supplementary material available at 10.1038/s41598-024-84364-6.

## Introduction

Bottom otter trawl fisheries are among the most productive fisheries worldwide^1^; nonetheless, their multiple direct and impacts on marine ecosystems are increasingly being studied, debated and condemned^2^. The unintended catch of some species or sizes (generally called ‘bycatch’) that are discarded at sea for a variety of reasons – too small, damaged, inedible, of little or no commercial value, protected, under the legal size or exceeding the allowed quotas, is one of the most dramatic issues of bottom trawling, which has the highest discard rates over marine fisheries^3^. The situation is hardly different for the Mediterranean Sea, where discard rates are estimated to be 20–65% of the total catch^4,5^, with peaks of 80% of the trawl catch (in weight) consisting of species without commercial value^6^.

Discarding is considered a major issue for Mediterranean trawl fisheries management, since most of the discards are thrown dead at sea and thus their removal affects both the food chain^7^ and fisheries, as fishing mortality neither does result in any economic advantage, nor will benefit fishing activities in future years^8^. In an effort to reduce discards, the Landing Obligation (LO; EU Regulation, 1380/2013 ^9^) was introduced to prohibit discarding for all the catches of the regulated species reported in the EU Regulation, 1241/2019 ^10^. In this way, the LO encourages fishers to avoid areas or seasons characterized by large amounts of undersized/unwanted fish and to employ more selective gear^11^.

In Mediterranean bottom trawl fisheries, the avoidance of unintended catch while not significantly penalizing the commercial catch is challenging since (i) small specimens of several fish and cephalopod species are in strong demand in most countries^12^, (ii) being multi-species fisheries, a selectivity device can be appropriate for one species and unsuitable for several others^13^. Those fisheries are currently managed through closed areas and seasons, limitations of the fishing effort and technical restrictions^5^. In the last decades, the most significant improvements in trawl selectivity have been obtained by changing mesh geometry at the codend level^12^. However, the codends currently allowed i.e. made of 40 mm square or 50 mm diamond meshes^10^ are insufficiently size selective for many commercial species^6,12,14,15^.

Besides improving trawl selectivity by applying changes in the codend design, another promising solution resides in the use of sorting grids to exclude unwanted species and sizes from the trawl catch^16^. Among the different studies conducted on sorting grids in the Mediterranean, several have focused on a two-sections Juveniles’ Sorting Grid (JSG, hereafter) installed in the trawl net just before the codend i.e. in Spain^17–22^, Türkiye^23–25^ and Italy^26–29^. The JSG is made of (i) an upper section having narrow bar spacing to allow only the smaller specimens (ideally only the undersized individuals of the regulated species) to pass through the bars and reach an escape opening placed behind the grid; (ii) a lower section being a hole that guides larger animals (ideally the legal-sized individuals) towards the codend. Some technical differences between the abovementioned studies are observed, especially in terms of spacing between bars (10 to 25 mm), bar shape (round or square), grid dimension, shape, material (e.g. aluminium or plastic, rigid or flexible) and tilt angle, presence or absence of the funnel and associated codend type (e.g. square or diamond meshes with various sizes). Despite the differences, the use of the JSG generally showed a catch reduction of undersized individuals of targeted species, although a commercial loss at different degrees depending on the species was observed.

No information is available, in the above-mentioned studies, on the vitality of individuals escaping from the JSG, and, in general, the fate of escaped individuals from the selectivity devices is rarely addressed in Mediterranean selectivity studies^27^. However, the behavioural interaction between the fish and the gear/device, and in particular their probability of being alive after escapement is necessary to better understand the overall performance of any gear/device aimed at increasing trawl selectivity^30^.

Given these premises, a two-sections JSG was tested in a Mediterranean bottom trawl fishery (Central Adriatic Sea) with the aim to answer the following questions:

- What are the conditions (vitality at escapement) of individuals escaping from the trawl through the JSG?
- Does installing the JSG change the catch composition in the codend, if compared to a standard trawl?
- What is the efficiency of the JSG at excluding undersized individuals of commercial species while retaining legal-sized individuals, if compared to a standard trawl?

## Methods

### Grid specifications

**Fig. 1:**
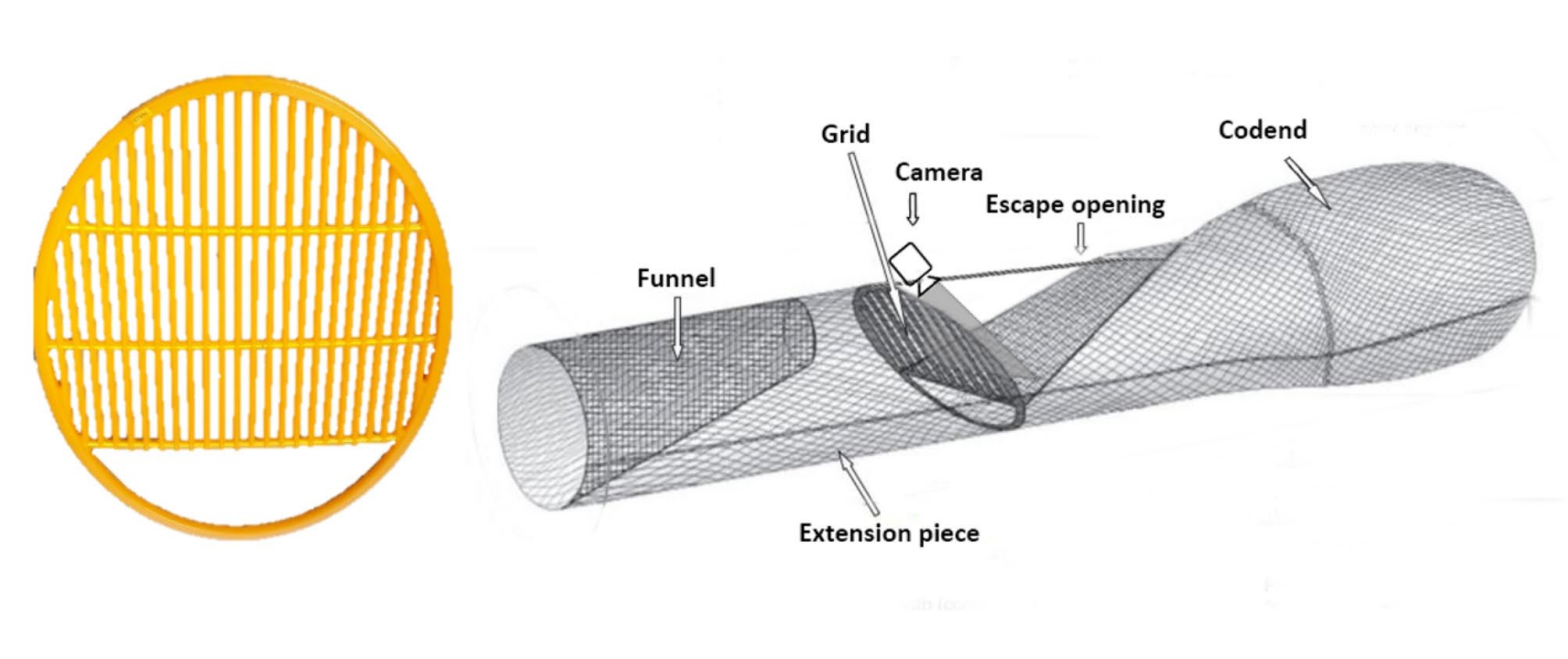
Left: Juveniles’ Sorting grid (JSG) selected for sea trials. Right: JSG installed in a Mediterranean trawl net, in the extension piece ahead the codend. (adapted from Bahamon et al.^17^); the position of the camera attached to the grid is reported.

The two-section JSG used in the sea trials was a light flexible grid (Flexgrid, Ocean Marine & Fishing Gear A/S, Denmark) made of an alloy of high-strength plastic material, which ensured elasticity and ability to both maintain a stiff configuration during trawling, and safely winding around the net winch during hauling^31^. The grid was 110 cm (height) x 85.6 cm (width), with (i) 3 horizontal bars (20 mm thick) spaced 25 cm from each other and 27 cm from the top and the bottom of the grid; (ii) 24 vertical bars (15 mm thick) spaced 20 mm from each other. The lower section of the grid (approximately 25% of the total area) consisted of a hole (see Sbrana et al.^27^ for grid scheme; Fig. 1, left).

The 20 mm bar spacing between vertical bars was selected to favour the escapement of undersized individuals of European hake (*Merluccius merluccius*, < 20 cm of minimum conservation reference size, MCRS^10^), which is the main target of Mediterranean demersal trawl fisheries^32,33^ and one of the species for which selectivity data of legal codends are most inadequate^6,12^. The 20 mm bar spacing was found to be the most promising at reducing the catches of undersized hakes without significantly losing the legal sized hakes in other areas^17–20,26,34^.

The grid was mounted on a 6 m long tubular netting section (polyethylene netting with 44 mm nominal mesh size) placed in the extension piece ahead the codend. An escape opening was cut into the upper portion of the net, just behind the sorting grid, and the two grid sections (upper and lower) were separated by a horizontal panel made of polyamide netting (20 mm nominal mesh size), not to allow escaped individuals from the bars to re-enter the trawl net. A guiding funnel (polyamide netting, 20 mm nominal mesh size) was installed before the grid to drive the fish towards the grid upper section, to maximize the contact probability with the bars (Figs. 1, right; S1). The funnel was an asymmetric cone, fixed to the upper part of the tubular netting section; its length was 2.80 m, and the distance of its rear part and the upper grid section was 20 cm.

### Selected area and fishery

The Pomo/Jabuka pits (GFCM Geographical Sub Area 17) are offshore fishing grounds with fine muddy sediments and 150–280 m depths, compared to the shallower waters (generally < 100 m) of the surrounding grounds of the GSA^35–37^. These particular physical features ensure a high productivity especially for Norway lobster (*Nephrops norvegicus*) and for European hake, which here has its main nursery ground, since juveniles concentrate in these deep muddy sea beds to feed on small crustaceans^38^. Both species are targeted by the bottom trawl fishery, together with other valuable species such as deep-water rose shrimp (*Parapenaeus longirostris*), horned octopus (*Eledone* spp.), monkfish (*Lophius budegassa*) and other species^5^. The Recommendation GFCM/41/2017/319 established the Jabuka/Pomo Pit Fisheries Restricted Area (FRA), divided in a core area, where any fishing activity is permanently prohibited, and a buffer area where only authorized vessels from Italy and Croatia (currently around 140) can operate, with temporal closures^39^. All the sea trials were conducted in the buffer area.

### Sea trials

The sea trials were conducted in Spring 2023, which is the best season to maximize the probability of catching small individuals (recruits and juveniles) of European hake in the area^40^, thus representing the best opportunity to test the JSG performances.

The first sea trial took place onboard the fishing vessel “Marcantonio II” from the harbour of San Benedetto del Tronto (Central Adriatic Sea, Italy), with length overall (LOA) of 19.96 m, gross tonnage of 91 GT and main power of 513 Kw. The gear used is the most common bottom trawl net type commercially employed in the area, called ‘Americana’^41^, in which the tubular netting section with the grid (described in 2.1) was inserted in the extension piece before the codend. No technical information of the net and rigging employed is here provided, since the first sea trial was exclusively focused on obtaining underwater footage of the JSG to monitor the vitality of escaped fish.

An easy-handling underwater camera (Go-Pro Hero 9 Black; GoPro^®^ Inc., California) associated with an aluminium deep-water housing (T-Housing H10 Energy; ActionPro^®^, Germany) was used to record footage down to 250 m of depth and, thanks to the housing additional space for auxiliary battery, cover the entire duration of commercial hauls i.e. up to two hours. White LED lights (Wurkkos^®^, DL40; ShenZhen Wokesida Technology Co., Ltd., China) at maximum intensity were added to illuminate the camera field. Although white light is known to affect fish behaviour more than red light^42–45^, it was here selected to prioritize the collection of clear footage, to allow identifying the species of individuals passing through the grid bars and estimate their vitality at escapement. Different positions of camera and lights were preliminarily tested in the sea trials. However, the setup that allowed to collect the data of the present study consisted in both camera and lights attached to the upper JSG section, behind the grid and facing downwards, to gain view of the grid bars and the escape opening (Fig. 1, right). The lights were placed to the camera’s sides and spaced around 40 cm apart. The light angle was selected to illuminate the camera field with minimal overlap to reduce ‘backscatter’ i.e. when lighting illuminates particles in the water between the camera and subjects (grid bars, fish), caused by turbidity expected when gear towing. Camera settings were 1080 pixels and 30 frames per second.

The hauls were carried out following the commercial fishing procedures in terms of haul duration (range: 1–2 h) and towing speed (range: 3–4 knots). After each haul, both GoPro and torches’ batteries were replaced and the underwater footage was downloaded.

**Fig. 2:**
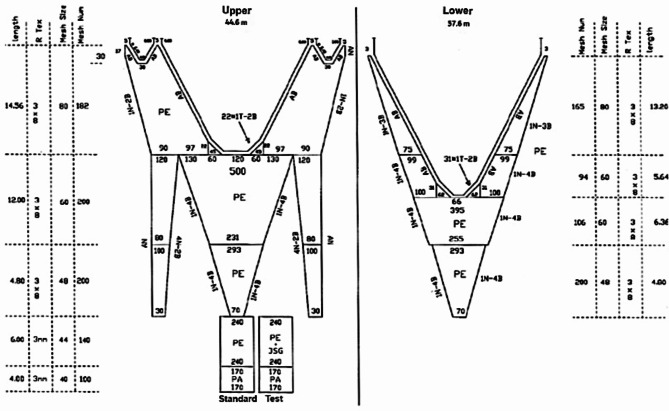
Scheme of the bottom trawl net (‘Americana’ net) used in the second sea trial with details of the extension piece plus codend of the two net configurations tested (Standard, Test).

The second sea trial took place on board the research vessel “G. Dallaporta” (length over all of 35.30 m, gross tonnage of 285 GT and main power of 810 Kw). The net used was an ‘Americana’ net type i.e. an asymmetrical 4-faces net with 520 meshes in the upper panel at the footrope level, a 44.6 m long headrope and a 57.6 m long footrope (Fig. 2). A single pair of stainless steel otter boards (180 × 110 cm, 350 kg each) was used to maintain the net horizontal opening. The otter boards were attached to the trawl by means of 80 m long sweeps and 15 m long bridles. A 54.6 m long tickler chain (Ø 7 mm) was employed, as it is commercially used with 4-faces nets towed in muddy bottoms to increase catch efficiency.

An alternate haul method was employed. Two identical configurations i.e. standard and test, differing only regarding the presence of the JSG installed in the extension piece of the test, were alternately mounted on the same trawl (Fig. 2). The same 6 m long extension piece made of diamond meshes (240 meshes at circumference; 44 mm nominal mesh size) and 4 m long codend made of square meshes (170 meshes at circumference; 40 mm nominal mesh size and 42.5 ± 0.6 mm measured mesh size after 20 measurements in wet conditions with the OMEGA mesh gauge;^46^) were used in both standard and test configurations (Fig. 2).

Haul duration was maintained at 70–75 min, and all hauls were performed in daylight at an average towing speed of 3.7 knots (range 3.5–3.8 knots). Hauls made with one trawl configuration were repeated with the other configuration, trying to replicate the track made during the previous haul but maintaining a distance of at least 250 m, to avoid tracks overlapping. Test hauls included the use of the same camera system and procedures described for the first sea trial, and in selected hauls a Data Storage Tags sensor (DST, Star Oddi^®^ hf, Iceland) was mounted on the JSG to collect data on its tilt angle. The gear performance (horizontal and vertical net openings) was monitored using acoustic sensors (PX MultiSensor, SIMRAD^®^, Norway).

After each haul, the catch was sorted by the scientific staff on board and divided by species. Then, the total abundance (number of individuals) and weight by species (or the lowest taxonomic level possible) were recorded. Furthermore, individual length measurements (total length for fish, mantle length for cephalopods and carapace length for crustaceans) were taken to the lowest 0.5 cm for all the commercial species retained in the codend. Sub-sampling was avoided.

### Data analysis

The Kruskal–Wallis H test (χ^2^) was first applied to test for differences between the wing horizontal openings and vertical net openings of the two configurations tested. We checked the net geometry to be sure to have the same fishing effort between the trawls alternatively towed. This was preparatory to further analyses.

### Vitality estimation

The underwater data collected from both sea trials was analysed using BORIS^47^, a free software specifically developed to investigate animal behaviour. Out of the total video recordings, which always covered the entire haul duration, only the footage that provided a clear view of the grid bars and escape opening during towing were included in the analyses. The videos were analysed at 0.5x speed, which was further reduced in case of fast or multiple simultaneous escape events. Two observers independently performed the video analyses to control for observer bias and discuss any uncertain events in the footage.

Each escaped individual from the grid bars was identified and its vitality state was recorded. The specimens were assigned to a species or to the lowest taxonomic level possible, e.g. family or taxon. Three mutually exclusive vitality states were predefined: “certainly alive”, “certainly dead” and “condition unsure”. The category “certainly alive” included all the individuals actively escaping from the grid bars and/or showing, during their stay in the camera field, clear and vigorous body movements. For fish species, the active swimming was considered as the main proxy of their vitality. An exception was made for the lesser spotted dogfish (*Scyliorhinus canicula*), since some individuals apparently immobile and passively passing through the bars clearly showed a sort of defence mechanism, consisting in the rolling up on themselves (Fig. S2). For cephalopods, other than body movements, the ink emission was also used to label them as “certainly alive”. The tail flip escape behaviour was selected as the main proxy for crustacean vitality; however, the status “certainly alive” was assigned also to those individuals that only actively moved the walking legs. We did not account for the degree of vitality, as it is commonly done in captured individuals^48^ due to both the little time spent by an individual in the camera field (usually around 1 s) and the insufficient video resolution to observe eventual external injuries in details, which did not allow to score the vitality.

The “certainly dead” category was assigned to those individuals which were passively transported by the water current through the bar spacing and throughout the camera field, without any movement or sign of life. Some additional details were required to include individuals in this category, such as, in fish, absence of operculum movements plus stiffening of the body after death and/or open mouth and, in cephalopods, lack of mantle pigmentation. Dying individuals with amputations of vital parts of the body, i.e. head or body missing, were also included in “certainly dead” category, even if some slight movements were detected.

The individuals not fulfilling the criteria described above for both “certainly alive” and “certainly dead” categories, were assigned to the “condition unsure” category. This category included those individuals passively transported by the water current through the bar spacing, usually without any movement or sign of life, but for which there was no proof of death. A typical example of a “condition unsure” consisted in those individuals escaping from the grid bars and from the camera field too fast to be properly observed.

The data obtained from the video analysis was used to provide an estimation of the probability, independently for each species, of being alive right after escapement. Each individual was assigned a score, i.e. 0 for “certainly alive”, 1 for “condition unsure” and 2 for “certainly dead”. Then, we estimated the expected average value *P*_*q*_ i.e. the probability for a species to score *q*:

1 $$\:{P}_{q}=\frac{\sum_{j=1}^{h}\left\{\frac{1}{{n}_{j}}\sum_{t=1}^{{n}_{j}}equal(q,{k}_{jt})\right\}}{h}$$

*with*

$$\:equal\left(q,k\right)=\left\{\begin{array}{c}1\:\forall\:\:k=q\\\:0\:\forall\:\:k\ne\:q\end{array}\right\}$$

where *h* is the number of hauls conducted, n_*j*_ is the number of individuals given a score in haul *j* and *k*_*jt*_ is the score given to the individual *t* in haul *j*.

Also, we quantified the probability *Pm*_*q*_ for a species of obtaining a score that does not go above 1 i.e. the probability of not being “certainly dead”, as:

2 $$\:{Pm}_{q}=\frac{\sum_{j=1}^{h}\left\{\frac{1}{{n}_{j}}\sum_{t=1}^{{n}_{j}}lequal(q,{k}_{jt})\right\}}{h}$$

*with*

$$\:lequal\left(q,k\right)=\left\{\begin{array}{c}1\:\forall\:\:k\le\:q\\\:0\:\forall\:\:k>q\end{array}\right\}$$

The Efron percentile 95% Confidence Intervals (*CIs;*^49^) were used to estimate the uncertainty around the probability values, by applying a double bootstrap methodology with 1000 repetitions, following the procedure described in Brinkhof et al.^50^. The method described above incorporates the effect of potential between-haul variation in fish vitality and the uncertainty resulting from the limited sample sizes at single haul^51^.

Furthermore, the difference *∆*$\:{P}_{q}$ in vitality probability $\:{P}_{q}$ between species *x* and *y* was estimated by:

3 $$\:\varDelta\:{P}_{q}={{P}_{q}}_{y}-{{P}_{q}}_{x}$$

By applying the technique described in Herrmann et al.^52^, the *CIs* for Eq. 3 were obtained based on separate bootstrap populations for $\:{{P}_{q}}_{x}$ and $\:{{P}_{q}}_{y}$. The significance was detected by inspecting if the *CIs* contained the value 0.0. If the 0.0 value was within the *CIs*, no significant difference was detected. The analyses were performed using the statistical software SELNET^53^.

### Catch dominance

The catch dominance analysis was performed to evaluate if installing the JSG changed the codend catch composition compared with a standard trawl i.e. if the proportion of each species in the catch was significantly different between the two configurations tested (standard, test).

We assigned a fixed rank to each single species caught in the sea trials, by including it into one of the following 4 categories: (i) ‘Target species’, i.e. the main commercial species targeted by the Italian bottom trawl fishery in the Jabuka/Pomo Pit FRA^5^; (ii) ‘other species of commercial value’, i.e. additional species having commercial value; (iii) ‘Species of no commercial value’, i.e. those species usually discarded by fishers in the area; (iv) ‘Protected species’, i.e. those species included in EU regulations and International lists (e.g. EU Habitat directive, IUCN red list).

We estimated the average performance of the two nets (standard and JSG-equipped net) by averaging the catch dominance curves over hauls. The curves were estimated, in both number of individuals (*dn*_*i*_) and weight (*dw*_*i*_), for each configuration, by using the following Equations^6,41,54^:

4 $$\:{dn}_{i}=\sum\limits_{j=1}^{h}\left\{\frac{{n}_{ij}}{\sum_{i=1}^{S}{n}_{ij}}\right\}$$

5 $$\:{dw}_{i}=\sum\limits_{j=1}^{h}\left\{\frac{{\rho\:}_{ij}\times\:{n}_{ij}}{\sum_{i=1}^{S}\left\{{\rho\:}_{ij}\times\:{n}_{ij}\right\}}\right\}$$

where *j* represents the haul and *i* is the species rank previously defined. *n*_*ij*_ is the number of individuals of the species *i* being counted in haul *j.* Parameter $\:{\rho\:}_{ij}$ is the average weight of species *i* in haul *j* in a given fraction of the catch, and it is obtained from the total weight and number of individuals. *S* is the total number of species considered, whereas *h* is the total number of hauls conducted with the specific net configuration.

The cumulative dominance curves were then estimated, in both number of individuals (*Dn*_*I*_) and weight (*Dw*_*I*_), to better represent species dominance patterns, as follows:

6 $$\:{Dn}_{I}=\sum\limits_{j=1}^{h}\left\{\frac{\sum_{i=1}^{I}{n}_{ij}}{\sum\:_{i=1}^{S}{n}_{ij}}\right\}\:\text{w}\text{i}\text{t}\text{h}1\hspace{0.17em}\le\:\hspace{0.17em}I\:\le\:\:S$$

7 $$\:{Dw}_{I}=\sum\limits_{j=1}^{h}\left\{\frac{\sum_{i=1}^{I}\left\{{\rho\:}_{ij}\times\:{n}_{ij}\right\}}{\sum\:_{i=1}^{S}\left\{{\rho\:}_{ij}\times\:{n}_{ij}\right\}}\right\}\:\text{w}\text{i}\text{t}\text{h}\:1\hspace{0.17em}\le\:\hspace{0.17em}I\:\le\:\:S$$

where *I* is the species rank summed up to in the nominator.

The Efron percentile 95% *CIs* were again used to provide the uncertainty of the values of dominance patterns obtained, following the procedure described in Herrmann et al.^52^.

Furthermore, the difference *∆d* in species dominance *d* in the test (*x*) and standard (*y*) nets was estimated by:

8 $$\:\varDelta\:d={d}_{y}-{d}_{x}$$

The *CIs* for Eq. 8 were obtained based on separate bootstrap populations for *d*_*x*_ and *d*_*y*_, following the procedure described in Herrmann et al.^52^. If the *CIs* contained the 0.0. value, no significant difference was detected.

### Catch comparison

The statistical software SELNET^53^ was used to analyse the catch data from the second fishing trial. Data obtained from the most abundant commercial species was treated as unpaired catch comparison data^55^. The catch comparison and catch ratio analyses were performed to investigate the size-dependent effect on the catch efficiency of each species by using the JSG.

For each species independently, we assessed the relative length-dependent catch comparison rate (*CC*_*l*_) of shifting from one configuration to another, by using Eq. 1^55^:

9 $$\:{CC}_{l}=\frac{\sum\limits_{j=1}^{ht}{nt}_{lj}}{\sum\limits_{j=1}^{hb}{nb}_{lj}+\sum\limits_{j=1}^{ht}{nt}_{lj}}$$

where *nb*_*lj*_ and *nt*_*lj*_ are the number of fish of length *l* of a given species retained in haul *j* by the baseline (*b* i.e. the standard net) and test (*t* i.e. the JSG-equipped net) net, respectively. Parameters *hb* and *ht* represent the total number of hauls conducted with *b* and *t*, respectively.

We estimated the catch comparison rate *CC(l*,***v****)* experimentally expressed by Eq. 9, by minimizing the Expression 10 (corresponds to maximizing the likelihood for the observed experimental rates):

10 $$\:-\sum\limits_{l}\left\{\sum\limits_{j=1}^{ht}\left\{{nt}_{lj}\times\:ln\left[CC\left(l,\varvec{v}\right)\right]\right\}\:+\:\sum\limits_{j=1}^{hb}\left\{{nb}_{lj}\times\:ln[1.0-CC\left(l,\varvec{v}\right)]\right\}\right\}$$

where the outer summation is over the length classes *l* and the inner summation is over the hauls *ht* and *hb* in the experimental dataset. The ***v*** parameter describes the catch comparison curve defined by *CC(l*,***v****)*. The experimental *CC*_*l*_ was modelled by the function:

11 $$\:CC\left(l,\varvec{v}\right)=\frac{exp\left[f\left(l,{v}_{0},\dots\:,{v}_{k}\right)\right]}{1+exp\left[f\left(l,{v}_{0},\dots\:,{v}_{k}\right)\right]}$$

where *f* is a polynomial of order *k* with coefficients *v*_*0*_ to *v*_*k*_, such that *v* = (*v*_*0*_, …, *v*_*k*_); *f* was considered up to an order of 4. Leaving out one or more of the parameters *v*_*0*_…*v*_*4*_ yielded 31 additional candidate models for the catch comparison function *CC(l*,***v****).* We estimated the catch comparison rate, among these models, by using the multi-model inference to obtain a combined model^55,56^. We based the ability of the combined model to describe the experimental data on the *p*-value, calculated based on the ratio between the model deviance and the degrees of freedom (DOF;^55,57^). A *p*-value > 0.05 implies suitable fit statistics for the combined model to describe the experimental data sufficiently well. With poor fit statistics (*p*-value < 0.05 and deviance/DOF > > 1), the residuals were inspected to determine whether the results were due to structural problems when modelling the experimental data, or to overdispersion in the data^57^.

*CC(l*,***v****)* quantifies the probability that a fish of length *l* is retained by the JSG-equipped net, provided that it is retained in one of the two nets (standard and test net). A *CC(l*,***v****)* value of 0.5 implies the same probability, for a fish with a given length *l*, of being retained by either configurations. However, the results of *CC(l*,***v****)* do not provide a direct relative value of the catch efficiency between the test and the standard nets. Therefore, we used the catch ratio *CR(l*,***v****)*, since it provides such direct comparison and can be easily derived from *CC(l*,***v****)* following the equation:

12 $$\:CR\left(l,\varvec{v}\right)=\frac{hb\times\:CC\left(l,\varvec{v}\right)}{ht\times\:[1-CC\left(l,\varvec{v}\right)]}$$

In this case, *CR(l*,***v****)* = 1.0 means that the catch efficiency of both configurations is equal, while *CR(l*,***v****)* = 0.25 implies that the test net is catching only 25% of the fish of length *l* compared to the standard net.

We again estimated the Efron percentile 95% *CIs* to provide the uncertainty around the values of both the catch comparison and catch ratio curves, through a double bootstrapping method with 1000 bootstrap repetitions. By using this approach, following the description given in Lomeli^58^, both within and between haul variations were taken into account.

### Exploitation pattern indicators

The exploitation pattern indicators were applied to summarize the relative performance of the two nets tested for the species previously selected in the catch comparison and catch ratio analyses^6,59,60^. Regarding the species with MCRS, the undersized individuals were considered as discards, while for the analysed commercial species without MCRS, since all the individuals were landed, no discard ratios were estimated.

The average percentages, in number of individuals below (*nP-*) and above (*nP+*) the MCRS, or total individuals (*nP*) for those species without MCRS, retained by the test compared to the baseline net, were estimated as follows:

13 $$\:nP-=\frac{hb\times\:\sum_{j=1}^{ht}\sum\:_{l\:<\:MCRS}^{}{nT}_{jl}}{ht\times\:\sum_{j=1}^{hb}\sum_{l\:<\:MCRS}^{}{nB}_{jl}}$$

14 $$\:nP+=\frac{hb\times\:\sum_{j=1}^{ht}\sum\:_{l\:\ge\:\:MCRS}^{}{nT}_{jl}}{ht\times\:\sum_{j=1}^{hb}\sum_{l\:\ge\:\:MCRS}^{}{nB}_{jl}}$$

15 $$\:nP=\frac{hb\times\:\sum_{j=1}^{ht}{\sum}_{l\:}{nT}_{jl}}{ht\times\:\sum_{j=1}^{hb}{\sum}_{l\:}{nB}_{jl}}$$

where *nT*_*jl*_ and *nB*_*jl*_ represent the estimation made for the test and baseline nets, respectively. The summations of *j* and *l* in (13), (14) and (15) are over the hauls *ht* and *hb*, and length classes *l*, respectively. An indicator value of 100% means that the test net caught the same number of individuals below (*nP-*) or above (*nP+*) the MCRS, or of total individuals (*nP*), as the standard net. Indicator values of 50% and 150% imply that the test net caught 50% less and 50% more individuals (below or above MCRS, or total individuals) than the standard net, respectively.

Discard ratios in number of individuals were then estimated, for species with MCRS, for both the test (*nDRatioT*) and baseline (*nDRatioB*) nets, as follows:

16 $$\:nDRatioT=100\:\times\:\frac{\sum_{j=1}^{ht}{\sum}_{l<\:MCRS\:}{nT}_{jl}}{\sum_{j=1}^{ht}{\sum}_{l\:}{nT}_{jl}}$$

17 $$\:nDRatioB=100\:\times\:\frac{\sum_{j=1}^{hb}{\sum}_{l<\:MCRS\:}{nB}_{jl}}{\sum_{j=1}^{hb}{\sum}_{l\:}{nB}_{jl}}$$

A discard ratio of 50% means that 50% of the individuals caught are discard. Again, the 95% *CIs* for each indicator were estimated using the double bootstrap method (1000 bootstrap repetitions) as in previous sections.

### Ethical statement

The fishing trials have been authorised by the Italian Ministry of Agriculture, Food Sovereignty and Forests and subsequently by the Italian Coastguard. No other authorization or ethics board approval was required. The only protected species caught during sea trials is *Alosa fallax*, included in the list of Annexes II and V of animals requiring close protection under the European Council Directive 92/43/EEC of 21 May 1992 on the conservation of natural habitats and of wild fauna and flora. No information on animal welfare or on steps taken to mitigate fish suffering and methods of sacrifice is provided since the animals were not exposed to any additional stress other than that involved in commercial fishing practices. The experimental procedures were thus not subjected to the ARRIVE guidelines. This article does not contain any studies with human participants performed by any of the authors.

## Results

**Fig. 3:**
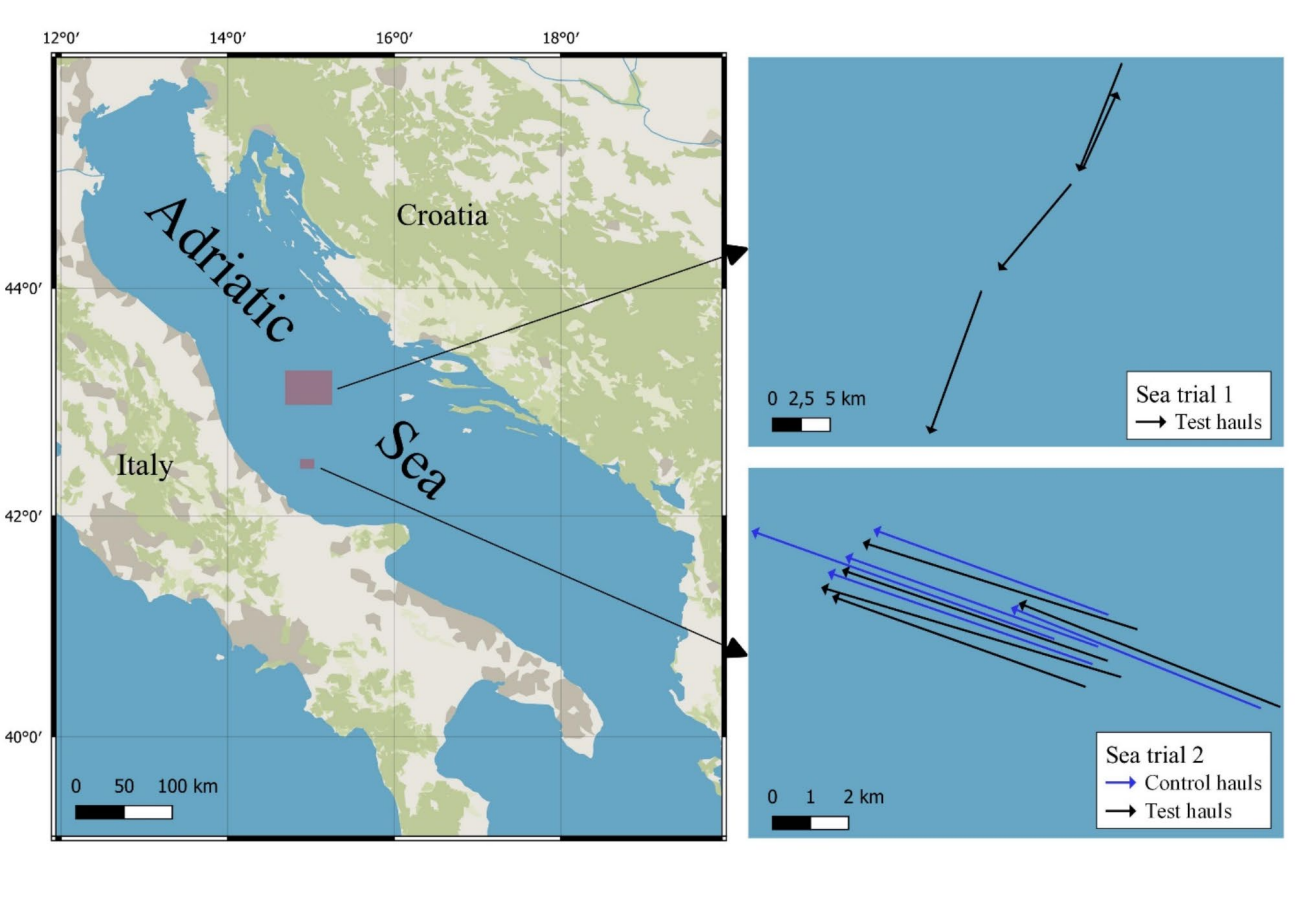
Map of the hauls conducted with the standard net (Control hauls) and the grid-equipped net (Test hauls) during the two sea trials, i.e. Sea trial 1 with fishing vessel and Sea trial 2 with research vessel.

A total of 14 hauls were conducted, 4 with the fishing vessel and 10 with the research vessel. Figure 3 maps their position and Table 1 lists further information, for each haul, on duration, mean depth, monitoring of net geometry, JSG inclination during towing and effective percentage of the video-monitored time.

**Table 1:** Hauls conducted during the two sea trials, with fishing and research vessels, respectively, information on net geometry and on the effective percentage of the video-monitored time for each haul is listed. Test: net equipped with the juveniles’ sorting Grid; Standard: standard net. SD: standard deviation.

	Haul	Net configuration	Haul duration [min]	Mean depth [m]	Mean wing horizontal opening [m ± SD]	Mean vertical net opening [m ± SD]	Grid inclination [° ± SD]	% of video-monitored time
Sea trial 1	1	Test	118	146.6	-	-	-	95.4
	2	Test	90	141.2	-	-	-	39.1
	3	Test	90	135.0	-	-	-	94.1
	4	Test	62	134.2	-	-	-	92.1
Sea trial 2	1	Standard	75	151.5	23.2 ± 0.6	1.2 ± 0.1	-	-
	2	Test	75	151.5	19.8 ± 3.0	1.8 ± 0.6	42.9 ± 2.5	40.3
	3	Test	75	151.5	18.4 ± 2.2	2.0 ± 0.4	45.6 ± 3.9	41.1
	4	Standard	75	151.5	17.2 ± 1.5	2.2 ± 0.3	-	-
	5	Standard	75	151.5	20.4 ± 1.8	1.8 ± 0.3	-	-
	6	Test	75	152.5	20.6 ± 2.3	1.6 ± 0.4	-	0.0
	7	Test	70	151.5	19.8 ± 0.5	1.8 ± 0.1	-	0.0
	8	Standard	70	152.5	22.3 ± 0.5	1.4 ± 0.1	-	-
	9	Standard	75	152.5	17.9 ± 2.3	2.0 ± 0.3	-	-
	10	Test	75	152.5	18.3 ± 2.2	2.0 ± 0.3	-	30.6

The K–W H test (χ^2^) revealed no significant differences in the net openings (m) between the standard and test nets (χ^2^ = 2.589, DOF = 1, *p*-value = 0.1076 for wing horizontal opening; χ^2^ = 1.5138, DOF = 1, *p*-value = 0.2186 for vertical net opening) monitored in the second sea trial.

### Vitality estimation

A total of 9 hauls were conducted with the JSG and camera system in both sea trials (Test hauls); the percentage of the successfully video-monitored time for each haul is listed in Table 1. The analysis of underwater footage of hauls 6 and 7 was not possible due to (i) technical issues in the camera system and (ii) high water turbidity encountered throughout the entire haul (Table 1).

We identified 28 species, 7 genera and 5 taxa (Table S1). Both genera and taxa were used to group those species for which a precise identification could be biased. For instance, the “Flatfish” category (i.e. *Pleuronectiformes* order) included the following species: *Arnoglossus laterna*,* Citharus linguatula*,* Lepidorhombus boscii*,* Lepidorhombus whiffiagonis*. The “Triglidae” family included: *Eutrigla gurnardus*,* Lepidotrigla cavillone*,* Trigla lyra*. Also, three larger groups namely “undefined cephalopod”, “undefined fish” and “undefined (general)” were created to include all the small individuals and/or juveniles frequently observed escaping from the grid bars, for which a precise identification was not possible. Table S1 also reports the total number of escaped individuals for each species/genus/taxon. In some occasions, especially with red mullets (*Mullus barbatus*), we observed individuals actively re-entering the grid bars after escapement. In those cases, we removed the escapement event from the dataset to avoid fish over counting. Also, we did not consider all the dead individuals being passively ejected from the grid bars before the actual start of the haul (i.e. when the towing warps were fully deployed, the trawl net touched the bottom and the speed was set for towing), since they referred to the previous haul (Figure S3).

Table 2 summarizes information on the main species observed in the underwater footage and their vitality probability. Red mullet had the highest number of individuals observed in the footage, followed by deep-water rose shrimp and European hake. Only three species (European hake, deep-water rose shrimp, broadtail shortfin squid, *Illex coindetii*) were observed in all the 7 successfully monitored hauls, while the others were observed in 4 to 6 hauls (Table 2). Regardless of the species, the probability for individuals to be “certainly alive” was significantly more than 65% on average. Red mullet had the highest probability of being certainly alive after escapement, which was, on average, more than 99%, followed by the *Triglidae* family (95% on average) and by the lesser spotted dogfish (92% on average). A high probability of being certainly alive at escapement was recorded also for deep-water rose shrimp (83% on average). Individuals of European hake and Norway lobster had a probability of being certainly alive included between 66 and 86% and between 25 and 78%, respectively. Table 2 also lists, for the same species, their probability of not being “certainly dead” i.e. by including the individuals for which an unknown vitality state was assigned. Regarding Norway lobster, the marked increase from 67 to 94% (on average) was related to the high number of individuals of this species assigned to the “condition unsure” category (score 1), since they usually made few active movements while passing through the grid bars thus hampering the assignment of a “certain” vitality category (score 0 or 2).

**Table 2:** List of the main species observed in the underwater footage. No of hauls: number of hauls in which the species was identified. No of individuals: total number of escaped individuals observed.

	No of hauls	No of escaped individuals	Probability of being “certainly alive” (%)	Probability of not being “certainly dead” (%)
*Merluccius merluccius*	7	623	75.71 (66.18–86.05)	87.19 (79.78–95.23)
*Parapenaeus longirostris*	7	1149	82.25 (72.69–87.97)	95.34 (92.32–97.32)
*Mullus barbatus*	6	1669	99.16 (98.45–99.77)	99.70 (99.19–100.00)
*Eledone* spp.	6	34	75.00 (52.00-93.75)	93.75 (79.31–100.00)
*Illex coindetii*	7	172	65.45 (52.38-75.00)	85.45 (75.76–92.44)
*Nephrops norvegicus*	4	118	67.26 (25.00-78.33)	93.81 (88.50–100.00)
*Scyliorhinus canicula*	4	100	91.92 (82.50-96.97)	98.99 (95.45–100.00)
Flatfish	6	204	74.50 (65.67–84.04)	83.50 (75.63–91.60)
Triglidae	4	59	94.92 (85.00-100.00)	100.00 (100.00-100.00)

Probability of being “certainly alive” (%): probability of the species of being alive at the escapement (mean values % with 95% confidence intervals). Probability of not being “certainly dead” (%): probability of the species of being alive at the escapement if including also all the individuals for which the “condition unsure” category was assigned to (mean values % with 95% confidence intervals).

Table 3 reports the differences in the probability of being “certainly alive” between the main species investigated i.e. European hake, deep-water rose shrimp, red mullet and Norway lobster. Red mullet had a significantly higher probability of being certainly alive at escapement than both European hake, Norway lobster and deep-water rose shrimp, since, in all cases, both lower and upper *CIs* are above the 0.0 value. No significant differences were observed in the other comparisons (Table 3).

**Table 3:** Differences in the estimated probability of being “certainly alive” between the main species investigated.

Mullus barbatus VS Merluccius merluccius	23.45 (13.19–33.50)
*Parapenaues longirostris* VS *Merluccius merluccius*	6.54 (-6.51–17.99)
*Nephrops norvegicus* VS *Merluccius merluccius*	-8.45 (-42.21–6.27)
*Mullus barbatus* VS *Parapenaeus longirostris*	**16.91 (9.37–24.46)**
*Mullus barbatus* VS *Nephrops norvegicus*	**31.90 (20.83–73.70)**
*Nephrops norvegicus* VS *Parapenaeus longirostris*	-15.00 (-52.53–0.05)

Values in bold indicate a significant difference.

Further observations, not included in the analysis and thus not supported by statistics, were repeatedly done in underwater footage of several hauls. European hakes usually came out of the bars in the lower part of the upper grid section, while both red mullets and deep-water rose shrimps did not display an escapement trend in the vertical section of the grid. Also, several individuals of deep-water rose shrimp, European hake and broadtail shortfin squid often came out of the bars simultaneously, highlighting a marked gregariousness of those species. The presence of individuals stuck in the grid bars was observed in all the hauls successfully monitored, thus clogging the grid at different degrees (Figure S4). The prolonged permanence of the individuals stuck in the bars was often found to produce external injuries or amputations and cause death. Several dead individuals (regardless of the species) usually occurred at the end of each haul, when the net was retrieved at great speed before being hauled.

### Catch dominance

Table 4 lists the 51 animal species caught in the second sea trial, which belonged to 5 higher taxa (*Osteichthyes* and *Condrichthyes; Crustacea Decapoda; Mollusca Cephalopoda; Cnidaria*). The ‘Target species’ category included 5 species: European hake, Norway lobster, deep-water rose shrimp, horned octopus and monkfish. The 16 species classified as ‘Other species of commercial value’ included fish (14 species) and cephalopods (2 species). Out of the 29 ‘Species of no commercial value’ 22 were fish species, 4 were crustaceans, 2 were cephalopods and 1 was cnidarian. The ‘Protected species’ category only included the twait shad (*Alosa fallax*) listed in Annexes II and V of the Habitats Directive as requiring close protection^61^.

**Table 4:**
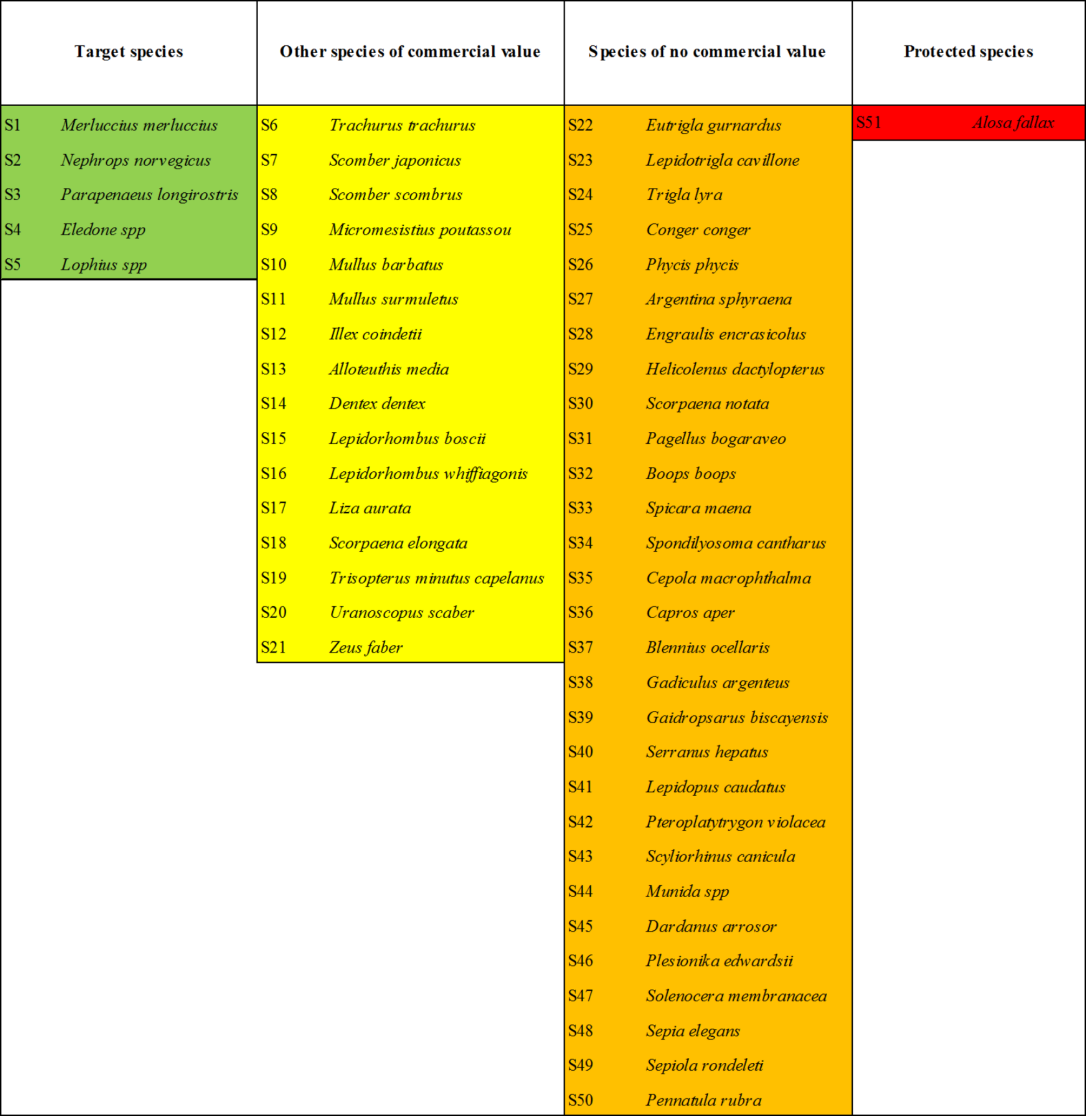
List of the animal species caught in the second sea trial with their assigned ranking (S).

**Fig. 4:**
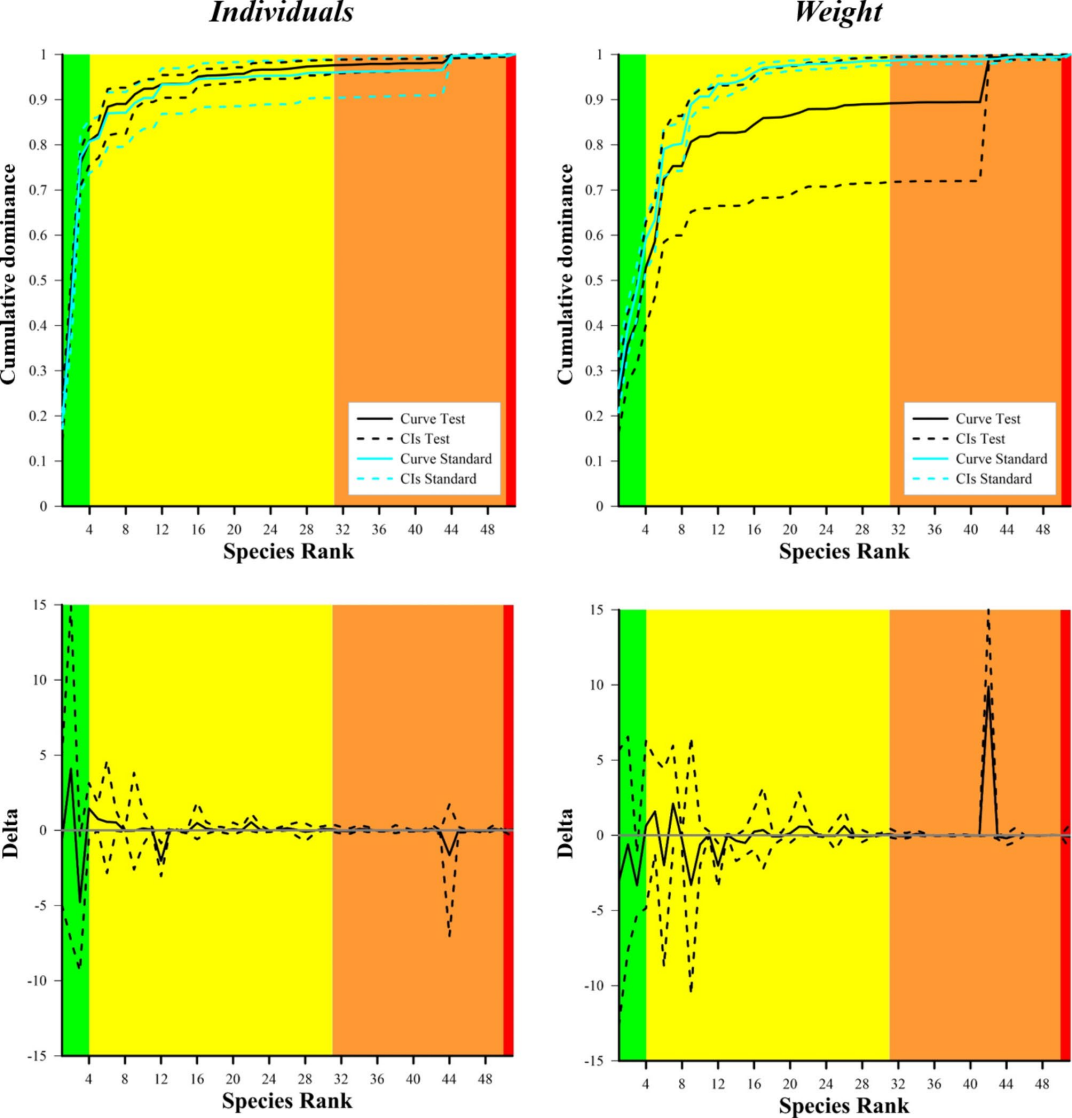
Top: Cumulative species dominance in the total catch of the test (black curve in solid line with 95% CIs in black dashed lines) and standard (light blue curve in solid line with 95% CIs in blue dashed lines) nets, in both number of individuals (left) and weight (right). Bottom: Delta plots resulting from the comparison of the species catch dominance (black curve in solid line with 95% CIs in black dashed lines) between the test and standard net, in both number of individuals (left) and weight (right). The 0 grey horizontal line represents an equal proportion between the two nets. In all the graphs, the green, yellow, orange and red areas represent the target species, the other species of commercial value, the species of no commercial value and the protected species, respectively.

Figure 4, top, shows the cumulative curves for the total catch of both the standard and JSG-equipped nets, in number of individuals and weight. In both nets, the target species (S1-S5) covered, on average, around 80% (in number of individuals) and 60% (in weight) of all the species caught. The same percentages increased up to 95% and 85–95% when including all the species having commercial interest (S1-S21). Therefore, the species with no commercial value represented only a minor proportion of the total catch. No significant differences between the curves of the two nets are reported, despite a higher increase, in weight, of the standard net curve compared to the test net curve at species S6 (Atlantic horse mackerel, *Trachurus trachurus*) and S9 (blue whiting, *Micromesistius poutassou*), and vice versa a higher increase of the test net curve compared to the standard net curve at species 42 (pelagic stingray, *Pteroplatytrygon violacea*). The latter was due to two individuals of *P. violacea* only caught by the test net (Fig. 4, top right and Table S2).

Figure 4, bottom, shows the delta plots resulting from the comparison of the catch dominance curves for the total catch, in both number of individuals and weight, and provides a detailed insight at each single species level. The *CIs* always contained the 0.0 value except for S3 (deep-water rose shrimp) and S12 (broadtail shortfin squid), indicating a lower proportion of their dominance in the catches of JSG-equipped net than in the catches of standard net, in both number of individuals (bottom left) and weight (bottom right).

### Catch comparison

**Fig. 5:**
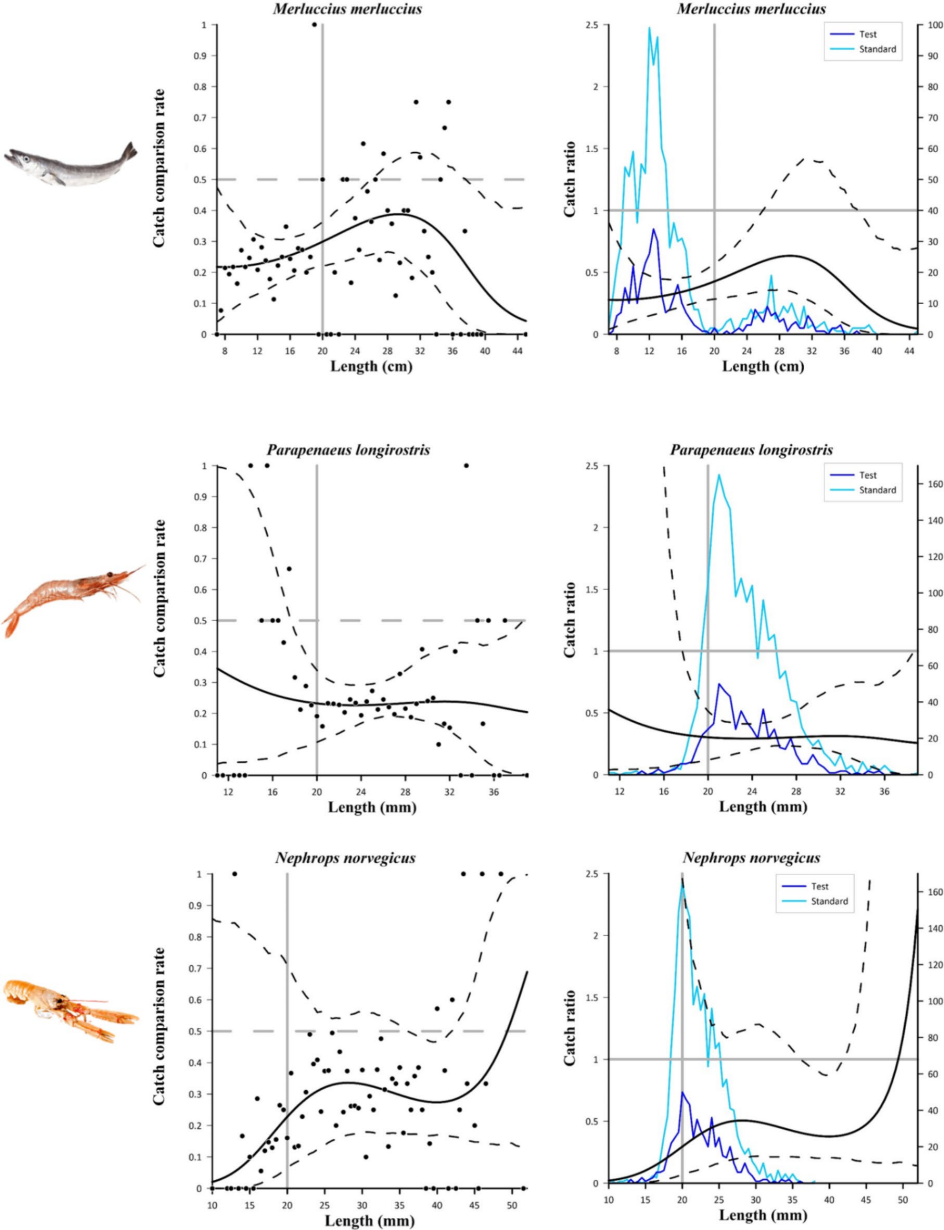
Results of the catch comparison analyses obtained for Merluccius merluccius (top), Parapenaeus longirostris (middle) and Nephrops norvegicus (bottom). The graphs on the left show the modelled catch comparison rate (black line) with 95% CI (black dashed curves); the black circles represent the experimental rate; the grey horizontal line at 0.5 represents the point at which both configurations have equal catch rates; the grey vertical lines represent the MCRS of the species. The graphs on the right show the catch ratio (black line) with 95% CI (black dashed curves); the blue lines represent the length frequency distributions obtained with the two trawls tested (test and standard); the grey horizontal line at 1.0 represents the point at which both nets have equal catch rates; the grey vertical line represents the MCRS of the species.

The catch data collected in the second sea trial allowed to perform the catch comparison analysis on six commercially important species, i.e. European hake, deep-water rose shrimp, Norway lobster, Atlantic horse mackerel, horned octopus, broadtail shortfin squid. The number of measured individuals for each haul is reported in Table S3. The fit statistics of the combined model used in the catch comparison analysis is reported in Table 5. We observed *p*-values ˂ 0.05 for deep-water rose shrimp and Norway lobster, but they were assumed to be due to overdispersion in the experimental rates, since the visual inspection of the modelled catch comparison curves against the experimental rates for these species did not indicate any length dependent patterns in the deviations (see Fig. 5).

**Fig. 6:**
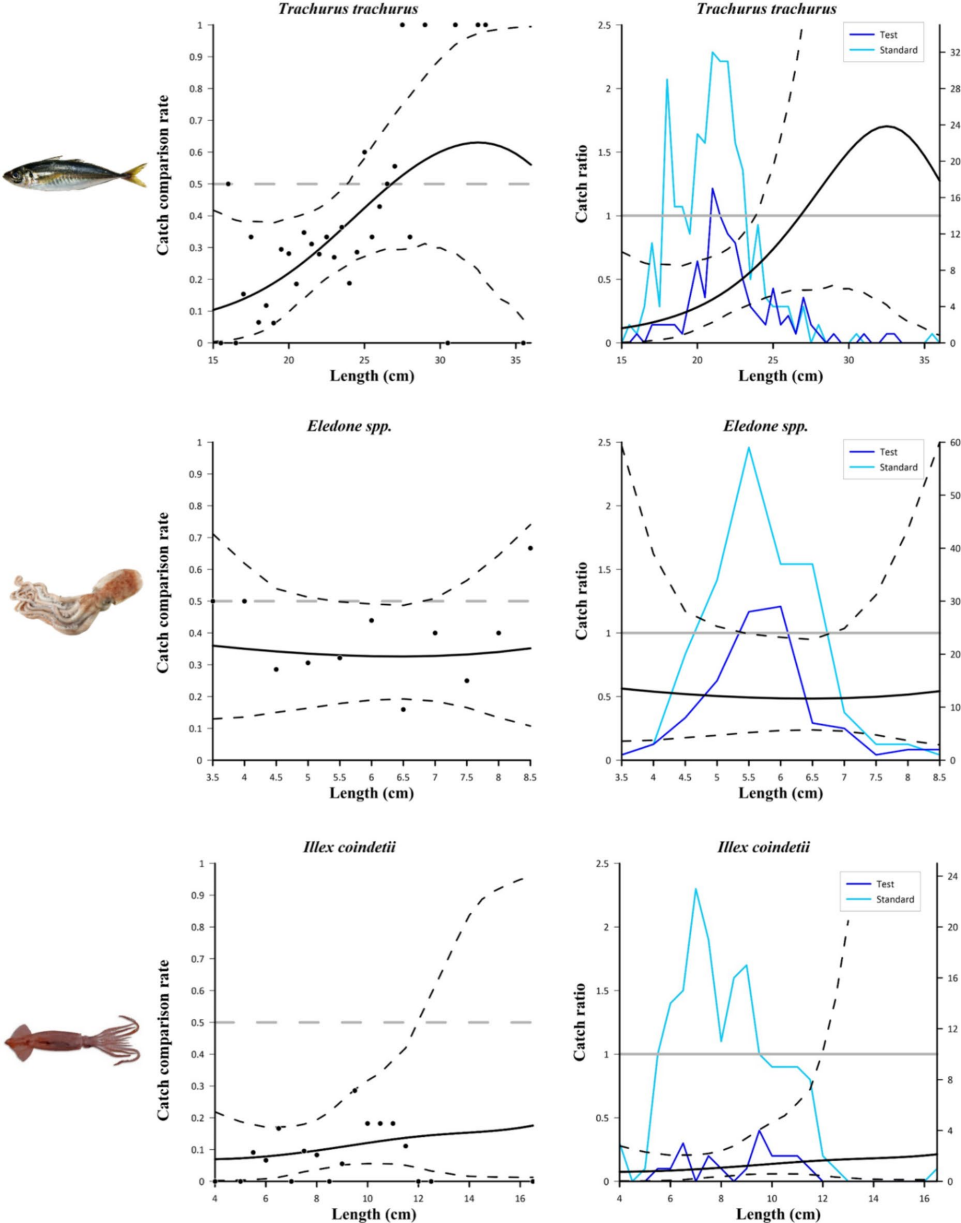
Results of the catch comparison analyses obtained for Trachurus trachurus (top), Eledone spp. (middle) and Illex coindetii (bottom). The graphs on the left show the modelled catch comparison rate (black line) with 95% CI (black dashed curves); the black circles represent the experimental rate; the grey horizontal line at 0.5 represents the point at which both configurations have equal catch rates. The graphs on the right show the catch ratio (black line) with 95% CI (black dashed curves); the blue lines represent the length frequency distributions obtained with the two trawls tested (test and standard); the grey horizontal line at 1.0 represents the point at which both nets have equal catch rates.

The catch comparison and catch ratio results for the selected species are shown in Figs. 5 and 6. Figure 5 presents the results obtained for the three main target species. Regarding the European hake (Fig. 5, top), the JSG-equipped net had a significantly lower catch efficiency than the standard net from the 7 cm to the 26 cm length class, and the difference was particularly evident for the undersized individuals (< 20 cm), not only from the catch comparison and catch ratio curves, but also from the length frequency distributions obtained. Also, a significantly lower catch efficiency of the test net was observed from 38 to 45 cm, although only a few individuals of these length classes were caught by both nets (Fig. 5, top). A significantly lower retention of the JSG-equipped net compared to the standard net is observed also for deep-water rose shrimp, for almost all the length classes except for the smallest ones (11–18 mm), where the low number of individuals caught are reflected in the wide *CIs* of the curves and in a high dispersion of the experimental rates (Fig. 5, middle). The difference is particularly evident in the most represented length classes (18–28 mm; Fig. 5, middle). On the contrary, no significant differences in the catch efficiency of both nets are observed for Norway lobster except for the 36.5–41.5 mm length range, although the length frequency distributions from test net show a clear decrease compared to those of standard net, especially in the 16 to 30 cm length range (Fig. 5, bottom).

Same trends are observed for the Atlantic horse mackerel, horned octopus and broadtail shortfin squid (Fig. 6). Regarding the Atlantic horse mackerel, no individuals below the MCRS of the species (15 cm) were caught in the sea trial; the lower retention of the test net was significant from 15 to 23.5 cm (Fig. 6, top). In the curves of horned octopus, the difference is barely significant only from 5.5 to 6.5 cm i.e. where the *CIs* did not contain the horizontal line of both graphs representing the same catch efficiency (Fig. 6, middle). Regarding the broadtail shortfin squid, the JSG-equipped net was significantly less efficient than the standard net at catching individuals from 4 to 11.5 cm; in fact, the length frequency distribution of the test net shows a clear decrease within this length range, when compared to the standard net. (Fig. 6, bottom).

**Table 5:** Fit statistics of the combined model used in the catch comparison between the standard trawl and the JSG-equipped trawl. DOF: degrees of freedom.

	Merluccius merluccius	Parapenaeus longirostris	Nephrops norvegicus	Trachurus trachurus	Eledone spp.	Illex coindetii
*p*-value	0.2505	0.0202	< 0.001	0.2605	0.0587	0.1909
Deviance	68.03	68.94	131.63	31.26	13.29	17.19
DOF	61	47	66	27	6	13

### Exploitation pattern indicators

The exploitation pattern indicators, in number of individuals, are listed in Table 6. On average, the JSG-equipped net caught 30% (*nP-*; *CIs* 15.8–46.6%) undersized and 56% (*nP+*; *CIs* 33.8–94.8%) legal-sized European hakes compared to the standard net. The discard ratio of both nets revealed that 60–90% of hakes caught by both nets (75% and 85% on average for test and baseline net, respectively, without significant differences) were undersized (Table 6). Same results concerned the deep-water rose shrimp, with the test net catching significantly less undersized and legal sized individuals than the standard net. However, the discard ratio was lower than in European hake, ranging from 5 to 17% of the total catch, without significant differences between nets. The *nP +* values for Norway lobster and Atlantic horse mackerel and the *nP* values for horned octopus and broadtail shortfin squid again highlighted a significantly lower catch of the test net, if compared to the standard net (Table 6). For Norway lobster, the mean discard ratio obtained with the JSG-equipped net (7.8%) was less than the mean discard ratio obtained with the standard net (15.3%), although not statistically significant (the *CIs* of the two values overlap; Table 6). The absence of Atlantic horse mackerels below the MCRS caught by both nets, and the absence of MCRS for the two cephalopod species did not allow to estimate both *nP-* and discard ratio indicators (Table 6).

**Table 6:** Exploitation pattern indicators (values in average percentage with 95% confidence intervals) of the species selected for catch comparison analysis.

Species	Indicator	Mean % (95% CI)
*Merluccius merluccius* MCRS = 20 cm	*nP-*	**30.1 (15.8–46.6)**
	*nP+*	**55.9 (33.8–94.8)**
	*nDratioT*	75.1 (60.3–82.2)
	*nDratioB*	84.9 (79.7–89.1)
*Parapenaeus longirostris* MCRS = 20 mm	*nP-*	**40.5 (12.5–88.0)**
	*nP+*	**28.9 (19.8–41.9)**
	*nDratioT*	11.7 (4.7–17.3)
	*nDratioB*	8.6 (5.9–11.3)
*Nephrops norvegicus* MCRS = 20 mm	*nP-*	20.8 (4.8-508.3)
	*nP+*	**44.3 (19.4–87.0)**
	*nDratioT*	7.8 (3.8–11.3)
	*nDratioB*	15.3 (1.1–34.8)
*Trachurus trachurus* MCRS = 15 cm	*nP-*	*
	*nP+*	**38.6 (20.7–69.1)**
	*nDratioT*	0*
	*nDratioB*	0*
*Eledone spp.*	*nP*	**49.3 (24.6–95.8)**
NO MCRS		
*Illex coindetii*	*nP*	**11.2 (5.5–24.2)**
NO MCRS		

nP, (total), nP- (below the MCRS) and nP+ (above the MCRS) represent the number of individuals retained by the test net compared to the standard net. Values in bold indicate a significant difference between standard and JSG-equipped net. nDRatioT and nDRatioB represent the resulting discard ratios estimated for the test and baseline net, respectively. *: no individuals below the MCRS were caught.

## Discussion

There is an urgent need to find and propose technical solutions to reduce the discards produced by Mediterranean bottom trawl fisheries. On one hand, the presence of a large number of undersized specimens in the catches^12^ has significant effects on the population dynamics of the main demersal species, contributing to the overfishing of more than 90% of the analysed stocks in the basin^62,63^. On the other hand, the established LO of all the catches of species subjected to the MCRS in the Mediterranean is an issue for fishers, due to difficulties related to storing and bringing to land the former discard and to higher sorting time or labour needed^64^. The use of a juveniles’ sorting grid installed before the codend of bottom trawl nets is considered a promising solution, and has been promoted in recent research projects financed by the European Commission (e.g. MINOUW, IMPLEMED) and FAO (the present study project).

Installing a JSG in a bottom trawl is a concrete benefit if it manages to release undersized individuals alive. Knowing the conditions (vitality at escapement) of organisms is pivotal when evaluating the performance of any bycatch reduction device (BRD) aimed at increasing selectivity. The review by Kennelly and Broadhurst^16^ highlighted that sorting grids can improve the post-selection survival of juveniles compared to the codend meshes (especially diamond meshes), which tend to close during trawling. In several studies, grid-escaping fish were in fact observed to have minimal injury and stress^65–67^. Among Mediterranean selectivity studies, information on the vitality rates of the specimens escaping from selectivity devices is currently scarce, and absent in studies on JSG^27^. Here, we conducted a video analysis on the escapees from the JSG bars, and estimated the vitality probability immediately after escapement. We found that the majority of individuals, regardless of the species investigated, had a high probability of being alive immediately after escapement, with some species (e.g. red mullet, gurnards) significantly more than others (e.g. European hake, crustaceans). Therefore, the JSG tested is not likely to cause instant death after the passage through its bars. However, since the individuals were generally observed for around one second after escapement, they may have had direct or indirect consequences from passing through a trawl net and grid bars. For instance, physical injuries such as scale loss (in fish) and legs/antennas impairment or loss (in crustaceans) were sometimes observed in the footage. The energy spent by some individuals to find a way out (burst swimming, ink emissions in cephalopods etc.) could also affect their survival over a longer period of time. These capture-related stressors and injuries through contact with other fish, debris, or the gear itself were not assessed in the present study. To assess the long term survival rate of escaped fish from any selection device, further ad hoc studies are needed, such as those conducted on red mullet escaped from codends in the Aegean Sea^68–72^ and in the Strait of Sicily^73^.

We tested this sorting grid in a FRA where authorized trawlers usually catch large amounts of undersized individuals mainly of European hake^28,74^, and therefore additional management measures, besides temporal and spatial closures, are needed to contribute to the recovery of this stock^39^. In fact, the catch data obtained here on European hake with standard trawl and 40 mm square mesh codend confirms that the majority of individuals caught were undersized, leading to higher discard ratios (80–90% of the total catch in number) than in the shallower fishing grounds of the GSA 17, where the percentage of undersized hakes in the catch of a standard trawl with the same codend was, on average, 16–19% ^6,41^. On the contrary, taking into account the total catch, the commercial species represented 95% (in weight) and 85% (in numbers) of the all the species caught in the present study, which is the opposite to what observed in Petetta et al.^6^, where only 50% (in weight) and 20% (in numbers) of the total catch consisted of species with commercial value.

The installation of a JSG in the trawl net did not change the overall catch composition at the codend level, as demonstrated in the cumulative dominance curves between the two net configurations tested. However, significant differences were observed at the single species level. In Table 7, we summarized the results obtained for European hake, Norway lobster and deep-water rose shrimp by this and all the studies conducted in the Mediterranean on JSGs. Although there is a wide variability related to the different sampling methods applied, conditions (experimental and commercial), technical properties of the vessel, trawl net and grids tested and to the different areas and seasons, it is worth comparing these results. In the present study, the JSG-equipped net has shown to be effective in reducing the catch of undersized hakes (8–20 cm), although a significant loss was observed also for legal-sized specimens (20–26 cm). This is in line with the findings of Sbrana et al.^27^ that tested the same grid design and 40 mm square mesh codend in GSA 9. Similar outcomes are provided by previous studies conducted on JSG in GSAs 5 and 6 ^17–20,34^, where the 20 mm grid bar spacing allowed to significantly reduce the catches of undersized hakes, since the resulting length at first capture (L50) value was 17.2 cm (selection range, SR of 6.2 cm;^17^), 18.8 cm (SR of 4.1 cm;^18^) and 18.9 cm (SR of 3.4 cm;^34^). These L50 values, obtained in association with a 40 mm diamond mesh codend that is not legal anymore^10^, are in fact higher than those obtained with standard trawls and legal codends^12,14^. A 15 mm bar spacing was too narrow to avoid the catch of undersized hakes^19,34^, while a 25 mm bar spacing, tested in GSA 16, resulted in a significant loss of the marketable fraction (~ 40%^26^), which is however comparable to what found in the present study with 20 mm bar spacing (Table 7).

**Table 7:** Summary of all Mediterranean studies testing the juveniles’ sorting grid (JSG) and the results obtained, including the present study.

Species	GSA	JSG type	JSG bar spacing (mm)	Codend meshes (mm)	Sampling method	Results	Reference
*Merluccius merluccius*	6	rigid, with funnel	20	40, diamond	Double codend	L50: 18.8 cm; SR: 2.1 cm	Sardà et al.^18^
	6	rigid, with funnel	20	40, diamond	Double codend	L50: 14.2 cm; SR: 7.3 cm	Sardà et al.^20^
	6	rigid, with funnel	15	40, diamond	Double codend	L50: 5.9 cm; SR: 3.5 cm	Sardà et al.^19^
	6	rigid, with funnel	20	40, diamond	Double codend	L50: 13.3 cm; SR: 2.6 cm	Sardà et al.^19^
	6	flexible, with funnel	20	40, diamond	Cover-codend	L50: 17.2 cm; SR: 6.2 cm	Bahamon et al.^17^
	6	flexible, without funnel	20	40, diamond	Cover-codend	L50: 13.2 cm; SR: 3.6 cm	Bahamon et al.^17^
	5	flexible, with funnel	15	40, diamond	Divided bottom trawl	L50: 10.9 cm; SR: 5.1 cm	Massutì et al.^34^
	5	flexible, with funnel	20	40, diamond	Divided bottom trawl	L50: 18.9 cm; SR: 3.4 cm	Massutì et al.^34^
	22	rigid, with funnel	20	44, diamond	Cover-codend	Catch separation: 64% in number and 85% in weight	Aydin et al.^23^
	22	rigid, with funnel	20	40, square	Cover-codend	Catch separation: 83% in number and 93% in weight	Aydin et al.^23^
	22	rigid, with funnel	10–15	44, diamond	Double codend	Catch separation between 96 to 100% in weight	Aydin and Tosunoǧlu^24^
	16	rigid, with funnel	20	40, square	Alternate hauls with standard net	34% reduction in catch efficiency of undersized individuals; no reduction in catch efficiency of legal-sized individuals	Vitale et al.^26^
	16	rigid, with funnel	25	40, square	Alternate hauls with standard net	38% reduction in catch efficiency of undersized individuals; significant reduction (~ 40%) in catch efficiency of legal-sized individuals	Vitale et al.^26^
	16	rigid, with funnel	netting instead of bars − 40, square	40, square	Alternate hauls with standard net	44% reduction in catch efficiency of undersized individuals; 39% reduction in catch efficiency of legal-sized individuals	Vitale et al.^26^
	6	rigid, with funnel	netting instead of bars − 40, square	40, square	Alternate hauls with standard net	95% reduction in catch efficiency of undersized individuals	Maynou et al.^22^
	9	flexible, without funnel	20	40, square	Alternate hauls with standard net	Significant reduction in catch efficiency of undersized individuals; no reduction in catch efficiency of legal-sized individuals	Sbrana et al.^27^
	16	rigid, with funnel	netting instead of bars − 40, square	40, square	Alternate hauls with standard net	Significant reduction in catch efficiency of undersized individuals; no reduction in catch efficiency of legal-sized individuals	Geraci et al.^29^
	17	flexible, with funnel	20	40, square	Alternate hauls with standard net	70% reduction in catch efficiency of undersized individuals; 44% reduction in catch efficiency of legal-sized individuals	Present study
*Nephrops norvegicus*	6	flexible, without funnel	20	40, diamond	Cover-codend	L50: 20.5 cm; SR: 9.3 cm	Bahamon et al.^17^
	5	flexible, with funnel	15	40, diamond	Divided bottom trawl	L50: 21.2 cm; SR: 6.7 cm	Massutì et al.^21^
	5	flexible, with funnel	20	40, diamond	Divided bottom trawl	L50: 23.8 cm; SR: 8.6 cm	Massutì et al.^21^
	17	flexible, with funnel	20	40, square	Alternate hauls with standard net	79% reduction in catch efficiency of undersized individuals; 56% reduction in catch efficiency of legal-sized individuals	Present study
*Parapenaeus longirostris*	22	rigid, with funnel	10	44, diamond	Double codend	Catch separation 60.8% in number and 70.7% in weigth	Aydin and Tosunoǧlu^24^
	22	rigid, with funnel	15	44, diamond	Double codend	Catch separation 37% in number and 44.4% in weigth	Aydin and Tosunoǧlu^24^
	16	rigid, with funnel	20	40, square	Alternate hauls with standard net	59% reduction in catch efficiency of undersized individuals, 30% reduction in catch efficiency of legal-sized individuals	Vitale et al.^26^
	16	rigid, with funnel	25	40, square	Alternate hauls with standard net	73% reduction in catch efficiency of undersized individuals, 36% reduction in catch efficiency of legal-sized individuals	Vitale et al.^26^
	16	rigid, with funnel	netting instead of bars − 40, square	40, square	Alternate hauls with standard net	60% reduction in catch efficiency of undersized individuals; 25% reduction in catch efficiency of legal-sized individuals	Vitale et al.^26^
	6	rigid, with funnel	netting instead of bars − 40, square	40, square	Alternate hauls with standard net	100% reduction in catch efficiency of undersized individuals	Maynou et al.^22^
	9	flexible, without funnel	20	40, square	Alternate hauls with standard net	No reduction in catch efficiency of undersized individuals; significant reduction in catch efficiency of legal-sized individuals	Sbrana et al.^27^
	16	rigid, with funnel	netting instead of bars − 40, square	40, square	Alternate hauls with standard net	Significant reduction in catch efficiency of undersized individuals; significant increase in catch efficieny of legal-sized individuals	Geraci et al.^29^
	17	flexible, with funnel	20	40, square	Alternate hauls with standard net	59% reduction in catch efficiency of undersized individuals; 71% reduction in catch efficiency of legal-sized individuals	Present study

GSA: geographical sub area of the GFCM; L50: length at first capture; SR: selection range.

The JSG-equipped trawl, compared to the standard trawl, increased the size selectivity also for all the other species investigated in the present study, often for both the undersized and the commercial sizes. In particular, the catch loss for deep-water rose shrimp and broadtail shortfin squid was evident over their whole size range, following the findings by^26–28^ (Table 7). The results could hamper the diffusion of this BRD in the fishery, since the economic viability seems to be hardly ensured. In fact, the 20 mm bar spacing selected was probably too wide for small specimens that are in strong demand in most Mediterranean countries^75^, such as shrimps, cephalopods and small fish. Small technical modifications, such as a slight decrease in bar spacing of 2–3 mm, could help reducing the loss of legal-sized individuals of target species (e.g. European hake, Norway lobster and deep-water rose shrimp) without compromising the good results obtained on discard reduction. However, a JSG could be implemented in the fishing industry by providing, at least in the initial stages, economic compensation measures. Recoveries in the revenues are then expected in the medium and long term, as shown in bio-economic forecasting models^28,76^. A recent study investigated the fishers’ perception towards BRDs, including sorting grids^77^, highlighting their willingness to use and implement BRDs in commercial fisheries, given economic incentives and/or the possibility of a certified market e.g. ecolabel fish product using low impact gear. In this way, fishers could ensure a better price that compensates for the economic loss of some missed catches. Also, a combination of two or more selectivity devices (e.g. grid and codend technologies together) could help, in high multi-species fisheries, the discard reduction without significantly penalizing the revenues^16,78^. The use of any mitigation device improving selectivity and ensuring high survivability of the escapees should be then mandated in bottom trawl fisheries, especially in those areas with known high abundances of undersized fish, such as the Jabuka/Pomo Pit fishing grounds.

The JSG performance was probably affected by the presence, in a few hauls, of wooden logs and plastic debris not expelled from the grid, and of fish often observed stuck in the grid bars. By obstructing the grid surface, they may have reduced the grid filtering efficiency and the contact probability for some specimens, which could have passed directly through the lower section towards the codend. This clogging phenomenon usually hampers the size dependent escape process at bars’ level, as it is stated in previous studies on JSG^27,28,79^ or on the metal cage used in the Adriatic hydraulic dredge fishery^80^. Massutì et al.^34^ also detected a saturation problem at the JSG level probably due to a large amount of echinoderms and skates, which have caused a decrease in the escapement ratio. A correct grid inclination during fishing (i.e. with angle of attachment of 40–50°^23,24,81^, also observed in the present study) would help conveying big objects towards the lower section and then to the codend, but both (i) the presence of the funnel, which was necessary to make all the catch contact the bars of the upper section for being size selected, and (ii) the absence of escaping windows as in Turtle Excluder Devices (TEDs;^31,82^) sometimes hindered the deflection of big objects. Therefore, the JSG would be more efficient in those grounds with a lower abundance of debris and animals, such as in deeper waters e.g. 400–750 m^34^. The clogging of a large number of fish in the grid bars was found to be a significant cause of death and poor fish quality (presence of skin marks and injuries). This was probably due to the bars that, having a square-like shape due to mold requirements in the building of plastic grids, may have favoured the fish clogging. On the contrary, the use of round steel bars may help the fish deflection, but all the benefits obtained with a light plastic grid (easier to handle and cheaper) would be lost^81^.

In conclusion, the JSG tested was efficient at releasing most of the undersized individuals of commercial species alive. However, further investigations are needed to improve the catch performance for legal-sized individuals and reduce clogging issues e.g. by modifying the grid material, shape, inclination, the guiding funnel and/or the bar spacing. Also, a combination of sorting grid plus design changes at the codend level is suggested, to optimize trawl selectivity. In fact, the implementation of technical modifications in the trawl is likely the most feasible solution to reduce discards in the Jabuka/Pomo Pit FRA, in addition to the temporal and spatial closures already in place (Recommendation GFCM/41/2017/319). The use of alternative and more sustainable gears is also encouraged to safeguard juveniles and ensure the stocks’ rebuilding^83^; preliminary tests in this area have been conducted on baited pots, despite a poor catch efficiency was observed^35^.

## Electronic supplementary material

Below is the link to the electronic supplementary material.

Supplementary Material 1

## Data Availability

The data analysed in the present study are currently available for research purposes by contacting the corresponding authors.
