# Author Correction: Vitality insights of fish escaping from a sorting grid installed on a bottom trawl net

**DOI:** 10.1038/s41598-025-94407-1

**Published:** 2025-03-20

**Authors:** Andrea Petetta, Bent Herrmann, Daniel Li Veli, Giovanni Canduci, Ivan Tatone, Sara Bonanomi, Lorenzo Jacopo De Santis, Giordano Giuliani, Massimo Virgili, Paolo Carpentieri, Alessandro Lucchetti

**Affiliations:** 1 Institute for Marine Biological Resources and Biotechnology (IRBIM), National Research Council (CNR), Largo Fiera Della Pesca 2, 60125 Ancona, Italy; 2 The Arctic University of Norway UIT, Hansine Hansens Veg 18, 9019 Tromsø, Norway; 3 Fishing Gear Technology, SINTEF Ocean, Trondheim, Norway; 4 National Institute of Aquatic Resources, Technical University of Denmark, Hirtshals, Denmark; 5 CoNISMa, National Inter-University Consortium for Marine Science, Rome, Italy; 6 General Fisheries Commission for the Mediterranean (GFCM), Food and Agriculture Organization of the United Nations (FAO), Rome, Italy

Correction to: Scientific Reports 10.1038/s41598-024-84364-6, published online 02 January 2025

The Acknowledgements section in the original version of this Article was incomplete.

The authors are indebted to the crew of the F/V “Marcantonio II” and R/V “G. Dallaporta” for their valuable assistance in the field work. The views expressed in this publication are those of the author(s) and do not necessarily reflect the views or policies of the Food and Agriculture Organization of the United Nations.

now reads:

The authors are indebted to the crew of the F/V “Marcantonio II” and R/V “G. Dallaporta” for their valuable assistance in the field work. The views expressed in this publication are those of the author(s) and do not necessarily reflect the views or policies of the Food and Agriculture Organization of the United Nations. This study was conceived under the FAO Tender No. 2022/CSAPC/NFIGD/119841. This research project was also supported by the National Recovery and Resilience Plan (NRRP), Mission 4 Component 2 Investment 1.4—Call for tender No. 3138 of 16 December 2021, rectified by Decree n.3175 of 18 December 2021 of Italian Ministry of University and Research funded by the European Union—NextGenerationEU."

The original Article has been corrected.

