# Supplementary material for: Vitality insights of fish escaping from a sorting grid installed on a bottom trawl net
Source: Sci Rep. 2025 Jan 2;15:552. doi: 10.1038/s41598-024-84364-6 (PMC11696904; doi:10.1038/s41598-024-84364-6)
Supplement: Supplementary file 1 — Supplementary Material 1 [file 41598_2024_84364_MOESM1_ESM.docx]

**Supplementary Materials**


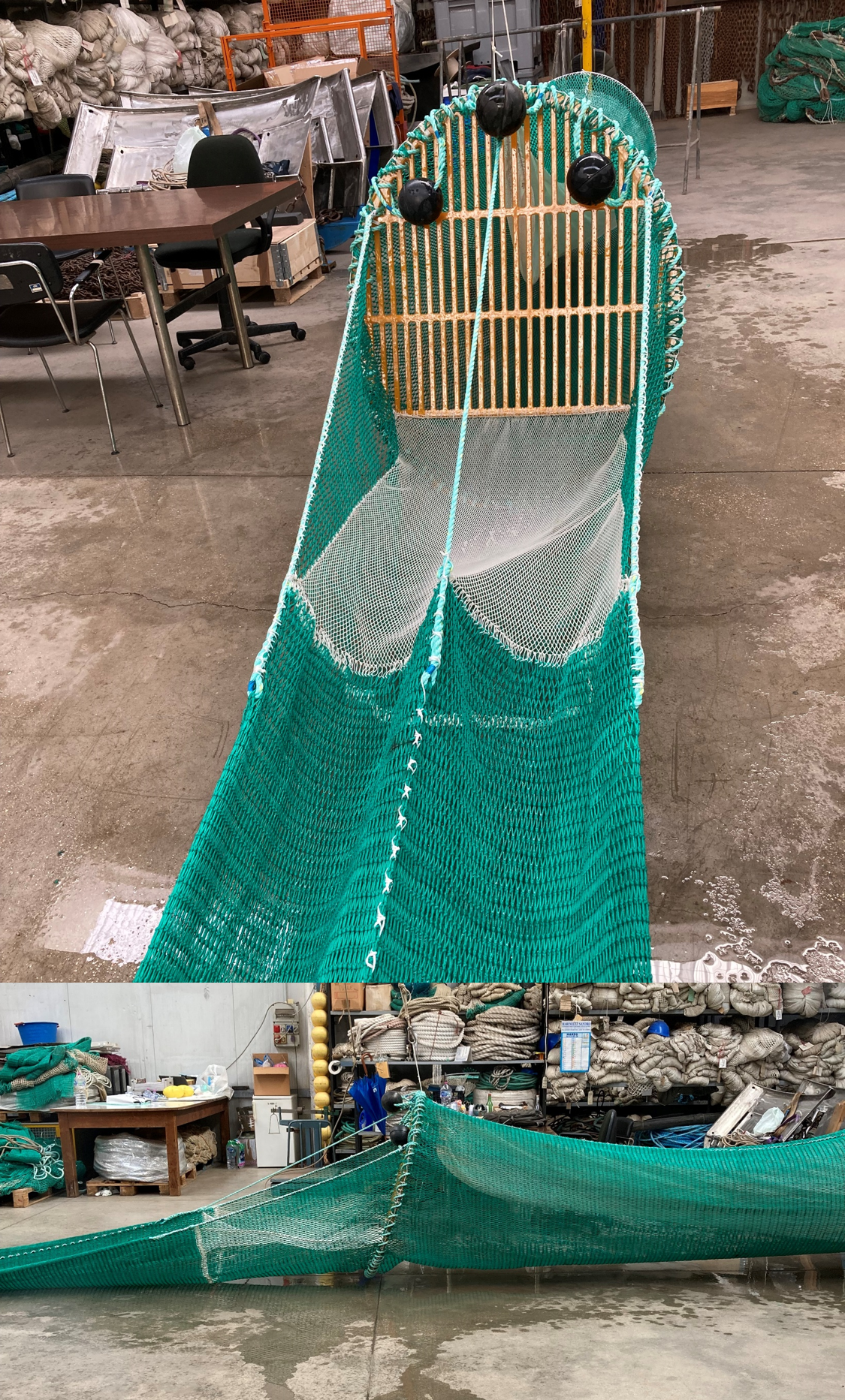


Figure S1. Front (top) and side (bottom) view of the Juveniles’ Sorting Grid (JSG) tested in the sea trials.


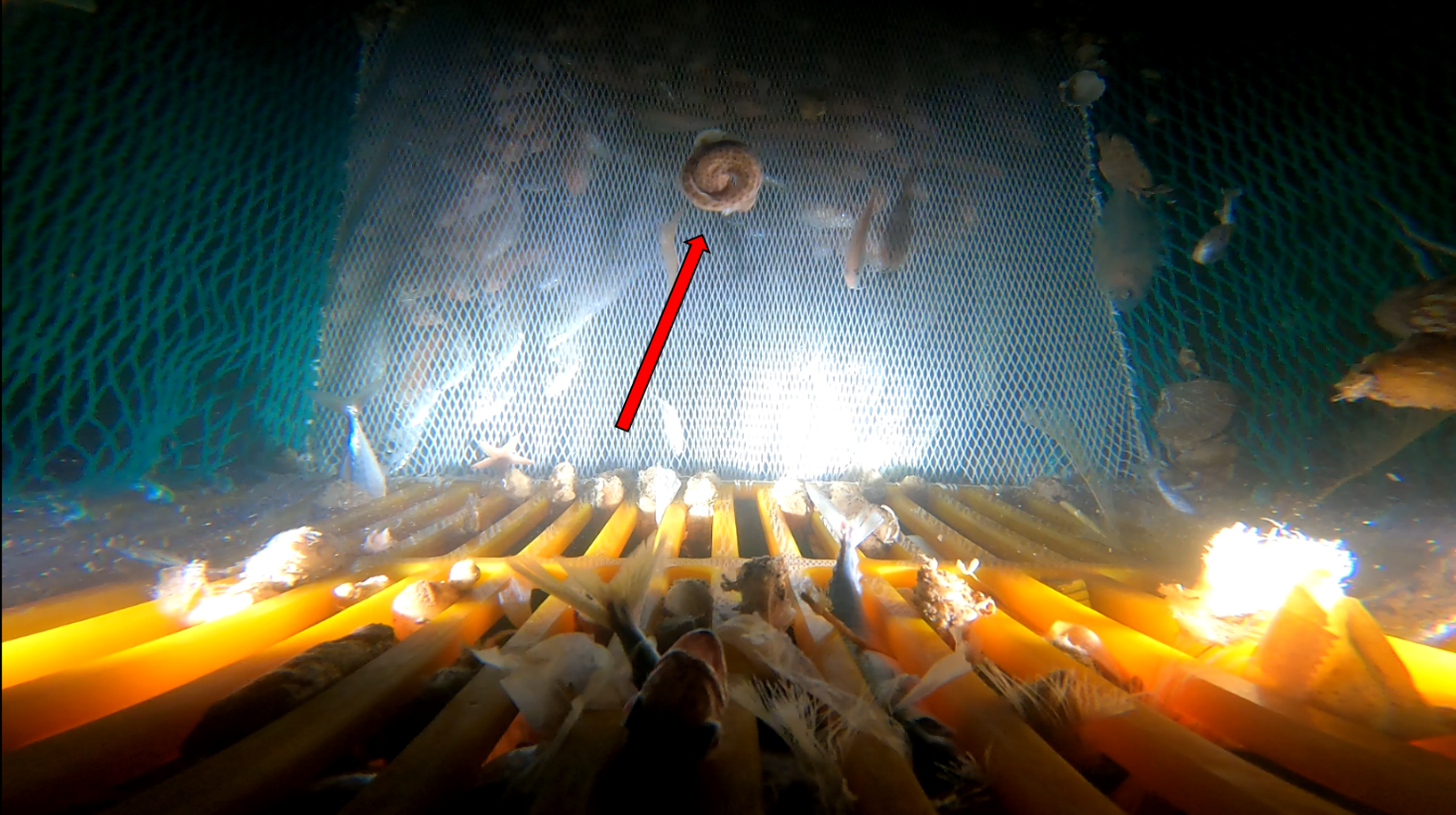


Figure S2. View of a particular behaviour of the lesser spotted dogfish, which rolls itself up and passively escapes through the grid bars. This individual was considered as "alive".


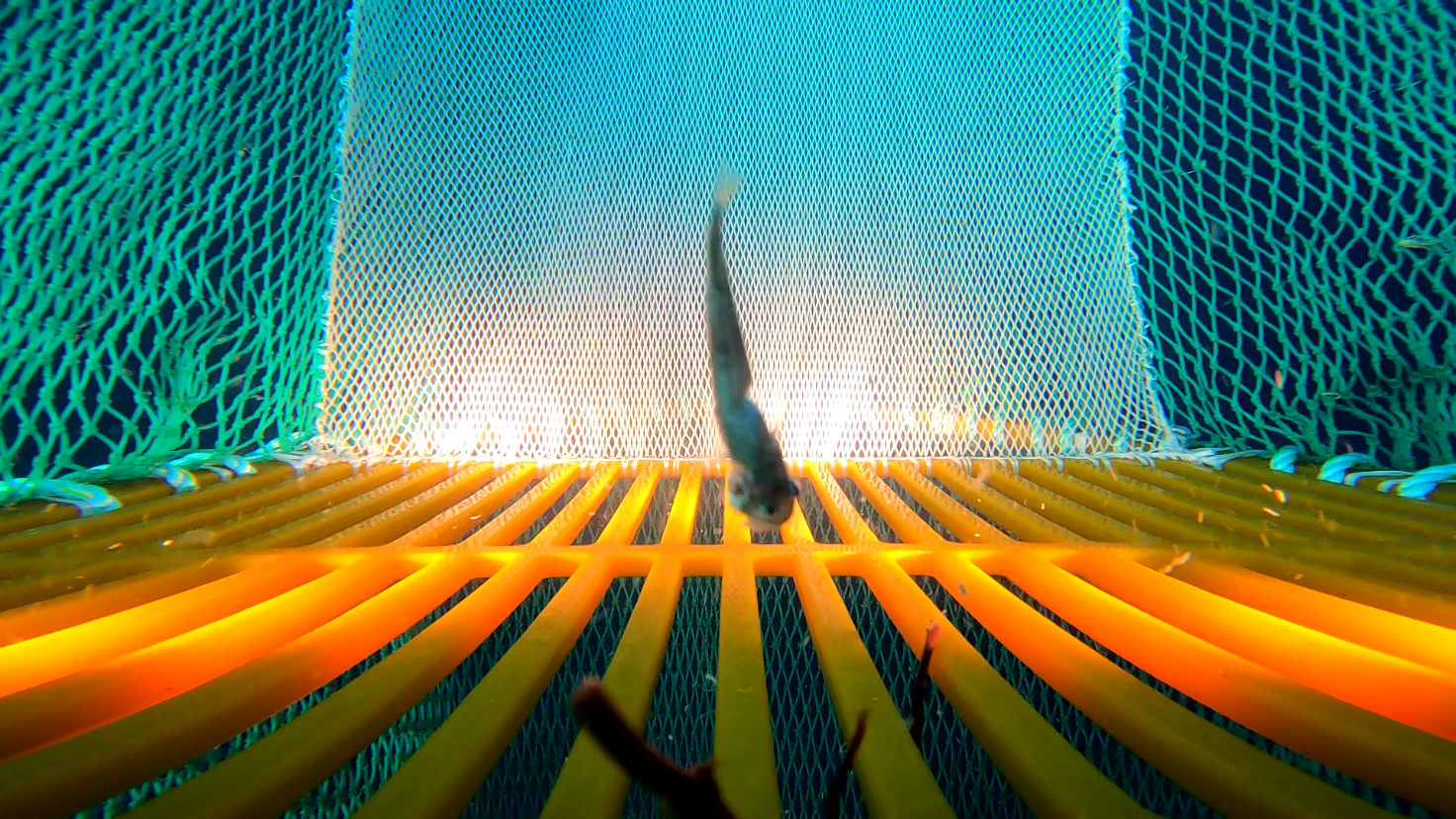


Figure S3. Example of a dead small hake passively going out through the grid bars prior to the actual haul start; this fish was not counted since it referred to the previous haul.


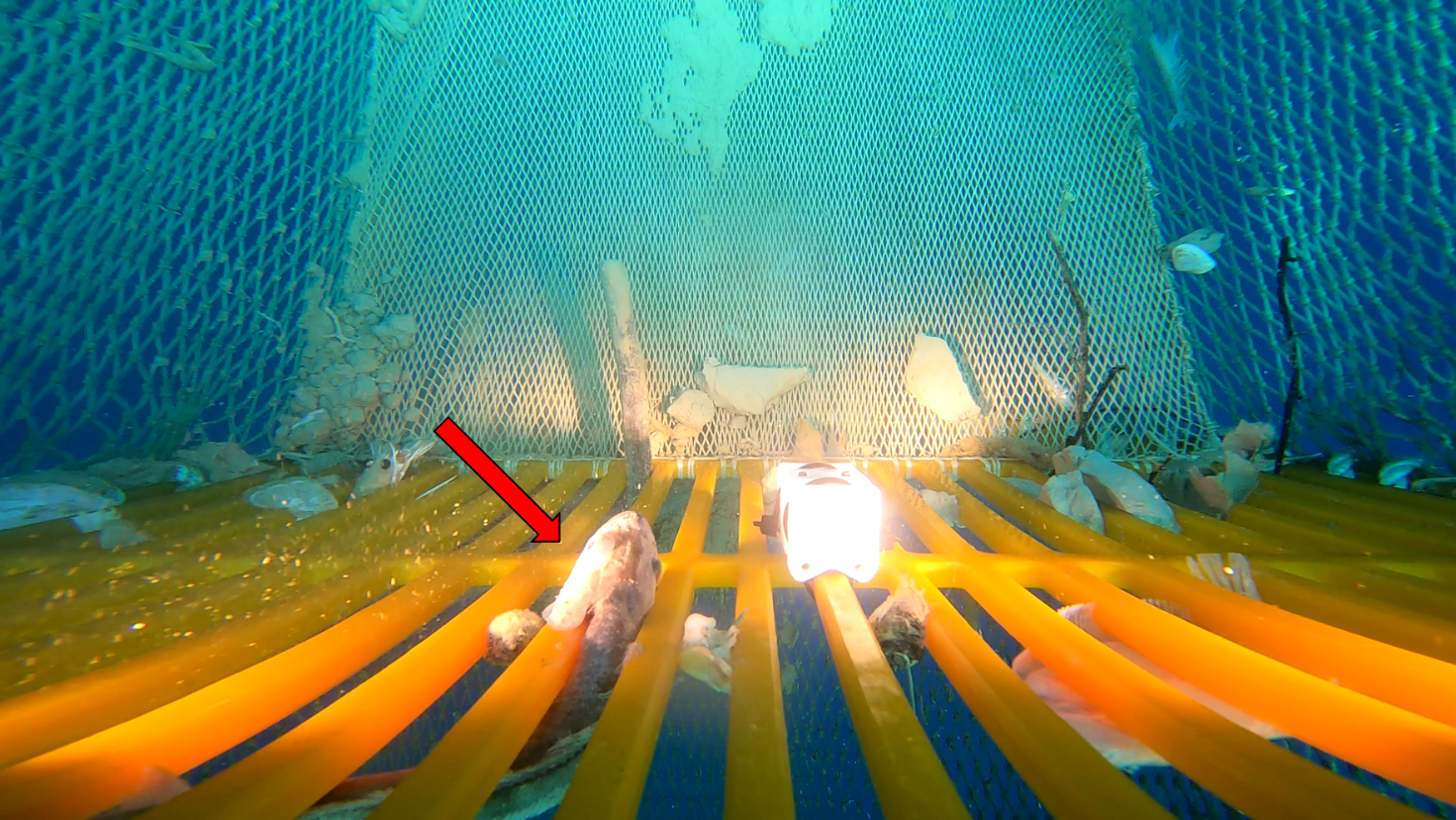


Figure S4. Detail of a European hake stuck between the grid bars for a long period of time (around 40 minutes!) before being ejected dead from the grid.

Table S1. List of the species, genera and taxa, with the associated total number of escaped individuals, observed in the underwater footages.

| **Species** | **No of escaped individuals** |
| --- | --- |
| *Alloteuthis media* | 43 |
| *Alosa fallax* | 1 |
| *Argentina sphyraena* | 7 |
| *Blennius ocellaris* | 4 |
| *Callionymus* spp. | 27 |
| *Cepola macrophthalma* | 15 |
| *Conger conger* | 4 |
| *Eledone* spp. | 34 |
| *Engraulis encrasicolus* | 4 |
| Flatfish | 204 |
| *Gadiculus argenteus* | 2 |
| *Illex coindetii* | 172 |
| *Lepidopus caudatus* | 1 |
| *Liocarcinus* spp. | 8 |
| *Lophius* spp. | 7 |
| *Merluccius merluccius* | 623 |
| *Micromesistius potassou* | 4 |
| *Mullus barbatus* | 1669 |
| *Mullus surmuletus* | 1 |
| *Munida* spp. | 28 |
| *Nephrops norvegicus* | 118 |
| *Parapenaeus longirostris* | 1149 |
| *Phycis phycis* | 1 |
| *Sardina pilchardus* | 14 |
| *Scomber* spp. | 6 |
| *Scyliorhinus canicula* | 100 |
| *Sepia elegans* | 3 |
| *Sepia officinalis* | 1 |
| *Sepiola rondeleti* | 5 |
| *Serranus hepatus* | 2 |
| Undefined cephalopod | 1404 |
| Undefined fish | 125 |
| Undefined (gen) | 102 |
| *Solenocera membranacea* | 2 |
| *Stichopus regalis* | 2 |
| Stomatopoda | 1 |
| *Todaropsis eblanae* | 2 |
| *Trachinus draco* | 1 |
| *Trachurus* spp. | 23 |
| Triglidae | 59 |
| *Zeus faber* | 1 |

Table S2. Dominance percentages (i.e. the proportion) of each species in the total catch of the JSG-equipped (test) net and the standard net, in both number of individuals and weight.

| **Category** | **Rank** | **Total catch Test** | | **Total catch Standard** | |
| --- | --- | --- | --- | --- | --- |
|  |  | **Individuals** | **Weight** | **Individuals** | **Weight** |
| Target  Species | S1 | 18.30 (14.72 - 23.61) | 23.07 (16.38 - 29.29) | 18.95 (17.20 - 20.80) | 26.11 (20.76 - 33.22) |
|  | S2 | 29.73 (20.04 - 35.71) | 12.70 (7.78 - 17.17) | 25.63 (16.15 - 32.16) | 13.30 (7.69 - 18.19) |
|  | S3 | 27.93 (25.50 - 31.31) | 5.02 (3.50 - 6.55) | 32.71 (30.20 - 36.88) | 8.34 (6.93 - 9.76) |
|  | S4 | 4.91 (3.60 - 6.11) | 11.97 (7.80 - 15.75) | 3.46 (2.23 - 4.72) | 11.33 (7.35 - 15.22) |
| Bycatch  Species  Of  Commercial  Value | S5 | 1.56 (0.89 - 2.54) | 5.80 (3.35 - 8.93) | 0.80 (0.55 - 1.23) | 4.21 (2.84 - 5.70) |
|  | S6 | 5.99 (3.52 - 9.44) | 13.75 (8.68 - 17.98) | 5.42 (3.52 - 7.75) | 15.74 (10.93 - 19.78) |
|  | S7 | 0.63 (0.10 - 1.40) | 3.01 (0.42 - 6.63) | 0.10 (0.00 - 0.25) | 0.91 (0.00 - 2.13) |
|  | S8 | 0 (0 - 0) | 0 (0 - 0) | 0.03 (0.00 - 0.10) | 0.32 (0.00 - 0.99) |
|  | S9 | 2.14 (0.623 - 5.95) | 5.36 (1.18 - 14.53) | 2.17 (1.22 - 4.05) | 8.66 (5.20 - 14.09) |
|  | S10 | 1.22 (0.47 - 2.24) | 1.17 (0.34 - 2.29) | 1.09 (0.63 - 1.89) | 1.783 (0.98 - 2.98) |
|  | S11 | 0.05 (0.00- 0.20) | 0.06 (0.00 - 0.26) | 0 (0 - 0) | 0 (0 - 0) |
|  | S12 | 0.97 (0.47 - 1.45) | 0.79 (0.27 - 1.51) | 3.00 (1.94 - 3.87) | 2.81 (1.53 - 4.04) |
|  | S13 | 0.05 (0.00 - 0.25) | 0.02 (0.00 - 0.08) | 0 (0 - 0) | 0 (0 - 0) |
|  | S14 | 0 (0 - 0) | 0 (0 - 0) | 0.02 (0.00 - 0.08) | 0.36 (0.00 - 1.71) |
|  | S15 | 0.10 (0.00 - 0.29) | 0.27 (0.00 - 0.99) | 0.15 (0.05 - 0.26) | 0.78 (0.22 - 1.52) |
|  | S16 | 1.51 (0.51 - 2.88) | 1.56 (0.54 - 3.14) | 1.02 (0.67 - 1.43) | 1.31 (0.86 - 1.82) |
|  | S17 | 0.24 (0.00 - 0.63) | 1.43 (0.00 - 3.79) | 0.12 (0.00 - 0.36) | 1.08 (0.00 - 3.20) |
|  | S18 | 0.05 (0.00 - 0.27) | 0.08 (0.00 - 0.43) | 0.02 (0.00 - 0.07) | 0.15 (0.00 - 0.74) |
|  | S19 | 0.10 (0.00 - 0.28) | 0.09 (0.00 - 0.26) | 0.10 (0.00 - 0.21) | 0.16 (0.00 - 0.35) |
|  | S20 | 0.19 (0.00 - 0.59) | 0.42 (0.00 - 1.23) | 0.12 (0.00 - 0.40) | 0.28 (0.00 - 0.71) |
|  | S21 | 0.05 (0.00 - 0.27) | 0.58 (0.00 - 2.86) | 0.02 (0.00 - 0.07) | 0.00 (0.00 - 0.01) |
|  | S22 | 0.78 (0.23 - 1.37) | 0.80 (0.26 - 1.38) | 0.24 (0.07 - 0.42) | 0.25 (0.06 - 0.46) |
|  | S23 | 0.15 (0.00 - 0.42) | 0.03 (0.00 - 0.09) | 0.10 (0.00 - 0.24) | 0.04 (0.00 - 0.09) |
|  | S24 | 0 (0 - 0) | 0 (0 - 0) | 0.02 (0.00 - 0.10) | 0.02 (0.00 - 0.12) |
|  | S25 | 0.05 (0.00 - 0.19) | 0.21 (0.00 - 0.95) | 0.02 (0.00 - 0.07) | 0.24 (0.00 - 1.01) |
|  | S26 | 0.15 (0.00 - 0.36) | 0.61 (0.00 - 1.64) | 0 (0 - 0) | 0 (0 - 0) |
|  | S27 | 0.24 (0.00 - 0.68) | 0.07 (0.00 - 0.22) | 0.20 (0.08 - 0.44) | 0.10 (0.04 - 0.20) |
|  | S28 | 0.24 (0.00 - 0.72) | 0.15 (0.00 - 0.48) | 0.34 (0.00 - 0.92) | 0.22 (0.00 - 0.57) |
|  | S29 | 0.10 (0.00 - 0.28) | 0.05 (0.00 - 0.16) | 0.12 (0.03 - 0.26) | 0.09 (0.02 - 0.17) |
|  | S30 | 0.10 (0.00 - 0.27) | 0.04 (0.00 - 0.12) | 0 (0 - 0) | 0 (0 - 0) |
|  | S31 | 0.10 (0.00 - 0.45) | 0.12 (0.00 - 0.53) | 0.03 (0.00 - 0.13) | 0.05 (0.00 - 0.16) |
| Species  Of  No  Commercial  Value | S32 | 0.05 (0.00 - 0.28) | 0.05 (0.00 - 0.26) | 0.09 (0.00 - 0.23) | 0.10 (0.00 - 0.28) |
|  | S33 | 0.05 (0.00 - 0.21) | 0.05 (0.00 - 0.23) | 0.10 (0.02 - 0.25) | 0.11 (0.01 - 0.21) |
|  | S34 | 0.10 (0.00 - 0.35) | 0.09 (0.00 - 0.32) | 0 (0 - 0) | 0 (0 - 0) |
|  | S35 | 0.10 (0.00 - 0.26) | 0.04 (0.00 - 0.13) | 0.05 (0.00 - 0.17) | 0.02 (0.00 - 0.07) |
|  | S36 | 0 (0 - 0) | 0 (0 - 0) | 0.02 (0.00 - 0.07) | 0.01 (0.00 - 0.04) |
|  | S37 | 0 (0 - 0) | 0 (0 - 0) | 0.02 (0.00 - 0.08) | 0.01 (0.00 - 0.03) |
|  | S38 | 0.10 (0.00 - 0.42) | 0.02 (0.00 - 0.07) | 0.09 (0.00 - 0.21) | 0.03 (0.09 - 0.07) |
|  | S39 | 0.05 (0.00 - 0.21) | 0.02 (0.00 - 0.09) | 0.03 (0.00 - 0.11) | 0.01 (0.00 - 0.04) |
|  | S40 | 0 (0 - 0) | 0 (0 - 0) | 0.02 (0.00 - 0.07) | 0.01 (0.00 - 0.04) |
|  | S41 | 0 (0 - 0) | 0 (0 - 0) | 0.02 (0.00 - 0.09) | 0.01 (0.00 - 0.05) |
|  | S42 | 0.10 (0.00 - 0.38) | 9.88 (0.00 - 27.64) | 0 (0 - 0) | 0 (0 - 0) |
|  | S43 | 0 (0 - 0) | 0 (0 - 0) | 0.12 (0.00 - 0.29) | 0.09 (0.00 - 0.24) |
|  | S44 | 1.46 (0.54 - 3.06) | 0.10 (0.04 - 0.21) | 3.10 (0.35 - 8.60) | 0.30 (0.04 - 0.75) |
|  | S45 | 0.05 (0.00 - 0.26) | 0.11 (0.00 - 0.61) | 0.03 (0.00 - 0.18) | 0.11 (0.00 - 0.51) |
|  | S46 | 0 (0 - 0) | 0 (0 - 0) | 0.03 (0.00 - 0.12) | 0.00 (0.00 - 0.01) |
|  | S47 | 0 (0 - 0) | 0 (0 - 0) | 0.02 (0.00 - 0.07) | 0.00 (0.00 - 0.01) |
|  | S48 | 0 (0 - 0) | 0 (0 - 0) | 0.05 (0.00 - 0.21) | 0.01 (0.00 - 0.03) |
|  | S49 | 0.10 (0.00 - 0.37) | 0.05 (0.00 - 0.02) | 0 (0 - 0) | 0 (0 - 0) |
|  | S50 | 0 (0 - 0) | 0 (0 - 0) | 0.02 (0.00 - 0.08) | 0.00 (0.00 - 0.01) |
| Protected  Species | S51 | 0.24 (0.00 - 0.53) | 0.45 (0.00 - 1.13) | 0.19 (0.04 - 0.47) | 0.54 (0.09 - 1.20) |

Table S3. Number of individuals of the most abundant commercial species measured in each haul, selected for the catch comparison analyses. No sub-samplings were performed.

| **Haul** | ***Merluccius merluccius*** | ***Parapenaeus longirostris*** | ***Nephrops norvegicus*** | ***Trachurus trachurus*** | ***Eledone spp.*** | ***Illex coindetii*** |
| --- | --- | --- | --- | --- | --- | --- |
| 1 | 260 | 463 | 423 | 110 | 65 | 45 |
| 2 | 68 | 133 | 172 | 38 | 26 | 5 |
| 3 | 94 | 160 | 237 | 39 | 36 | 8 |
| 4 | 286 | 455 | 561 | 71 | 22 | 52 |
| 5 | 174 | 417 | 260 | 43 | 39 | 17 |
| 6 | 37 | 52 | 26 | 26 | 10 | 1 |
| 7 | 116 | 139 | 117 | 15 | 20 | 4 |
| 8 | 262 | 375 | 168 | 35 | 59 | 52 |
| 9 | 129 | 208 | 91 | 59 | 18 | 10 |
| 10 | 61 | 90 | 59 | 5 | 9 | 2 |
